# Evaluation of Directed Causality Measures and Lag Estimations in Multivariate Time-Series

**DOI:** 10.3389/fnsys.2021.620338

**Published:** 2021-10-22

**Authors:** Jolan Heyse, Laurent Sheybani, Serge Vulliémoz, Pieter van Mierlo

**Affiliations:** ^1^Medical Image and Signal Processing (MEDISIP), Department of Electronics and Information Systems (ELIS), Ghent University, Ghent, Belgium; ^2^EEG and Epilepsy Unit, University Hospitals and Faculty of Medicine, University of Geneva, Geneva, Switzerland

**Keywords:** functional connectivity, lag estimation, information theory, multivariate time series, granger causality, ictal network, EEG connectivity

## Abstract

The detection of causal effects among simultaneous observations provides knowledge about the underlying network, and is a topic of interests in many scientific areas. Over the years different causality measures have been developed, each with their own advantages and disadvantages. However, an extensive evaluation study is missing. In this work we consider some of the best-known causality measures i.e., cross-correlation, (conditional) Granger causality index (CGCI), partial directed coherence (PDC), directed transfer function (DTF), and partial mutual information on mixed embedding (PMIME). To correct for noise-related spurious connections, each measure (except PMIME) is tested for statistical significance based on surrogate data. The performance of the causality metrics is evaluated on a set of simulation models with distinct characteristics, to assess how well they work in- as well as outside of their “comfort zone.” PDC and DTF perform best on systems with frequency-specific connections, while PMIME is the only one able to detect non-linear interactions. The varying performance depending on the system characteristics warrants the use of multiple measures and comparing their results to avoid errors. Furthermore, lags between coupled variables are inherent to real-world systems and could hold essential information on the network dynamics. They are however often not taken into account and we lack proper tools to estimate them. We propose three new methods for lag estimation in multivariate time series, based on autoregressive modelling and information theory. One of the autoregressive methods and the one based on information theory were able to reliably identify the correct lag value in different simulated systems. However, only the latter was able to maintain its performance in the case of non-linear interactions. As a clinical application, the same methods are also applied on an intracranial recording of an epileptic seizure. The combined knowledge from the causality measures and insights from the simulations, on how these measures perform under different circumstances and when to use which one, allow us to recreate a plausible network of the seizure propagation that supports previous observations of desynchronisation and synchronisation during seizure progression. The lag estimation results show absence of a relationship between connectivity strength and estimated lag values, which contradicts the line of thinking in connectivity shaped by the neuron doctrine.

## 1. Introduction

Many scientific fields are interested in detecting causal relationships between simultaneously observed signals, as they reveal the interplay between different processes and how they are linked within a larger system. One of the leading concepts for the detection of directional interactions, Granger causality, has been widely used in economics in an attempt to identify the driving and responding constituents within an economic environment (Pasquale, 2007; Beyzatlar et al., 2012; Plíhal, 2016). More recently, the same concepts have progressively entered the field of neuroscience and have lead to a new research field, referred to as “network neuroscience” (Seth et al., 2015; Hassan and Wendling, 2018). There is a growing body of evidence supporting the theory of large-scale networks of highly specialised and segregated areas within the brain. Within this context, the characterisation of functional brain networks in different normal and pathological states from neuroimaging data has become an exciting and promising field in brain research (Fornito et al., 2015; Bassett and Sporns, 2017). Even more recently, this concept has extended further into a new conceptual framework called “network physiology,” which focuses on the coordination and network interactions among diverse organ systems and subsystems as a hallmark of physiologic state and function (Bartsch et al., 2015).

Most of the functional connectivity measures seek to detect a statistical causation from the data, based on the theorem of Wiener, who proposed two criteria for causality: causes precede their effects, and causes should in some way predict their effects (Wiener and Masani, 1957). Granger adopted this definition of causality in 1969 to develop his famous index using predictions based on auto-regressive models (Granger, 1969). Over the years, a rich and growing body of literature has evolved both on the development of new metrics to quantify causal interactions and modifications of existing metrics, as well as practical implementations. With the help of information theory, measures capable of detecting non-linear interactions have been developed (Vicente et al., 2011; Montalto et al., 2014). The large number of available methods, often described with a large amount of technical detail, and variable choices of the relevant parameters often makes it difficult to choose and justify which method to use (Bressler and Seth, 2011; Bastos and Schoffelen, 2016). Several review papers have been published in an attempt to provide an overview, however the amount of comparative studies is limited and often focussed on a small subset of metrics and signal types (Pereda et al., 2005; Sakkalis, 2011; Wu et al., 2011; Silfverhuth et al., 2012; Fasoula et al., 2013; Olejarczyk et al., 2017; Siggiridou et al., 2019). With this study we want to help fill this gap by providing an extensive evaluation of several multivariate measures of directed causality on a wide range of simulation models, complementary to the work of Papana et al. (2013). In their work they evaluated a similar set of causality measures, looking also at the effect of time series length and coupling strength on the outcome. We extend this evaluation by adding the lag estimation and moving on from the simulations to apply the methods on real data.

One of the key postulations formulated in Weiner's criteria for causality is that the cause always precedes its effect. This seems to be a fair presumption and is in line with what we see in most real-life applications. Time delays between communicating variables are an inherent element of network systems and can have a large impact on its dynamics. Changing the delays of connections has been shown to affect synchrony in spike-burst simulations of neuronal networks (Jirsa, 2008). To gain a full understanding of the network processes that underly the observations, we therefore need estimations not only of the connections but also of their delays. These delays have been largely ignored up to now, partly because we lack proper tools to estimate them. To address this issue we propose three new methods to estimate the lags in multivariate time series and evaluate them on simulation models and a real-world application.

The paper is structured as follows: we begin with a mathematical description of the simulation models and causality measures that are to be evaluated in this study. We compare the performance of all possible combinations to gain a better understanding of the interplay between the system characteristics and the results from the functional connectivity measures. Then, we formulate the methods for lag estimation, based on autoregressive modelling and information theory, and also evaluate them on simulation models. In the end, we apply the same methods on a clinical dataset to see how we can translate our newly gained insights into real-world applications. The results are presented in section 6 and discussed in section 7. Finally, we end with some concluding remarks in section 8.

## 2. Simulation Models

**Figure 1:**
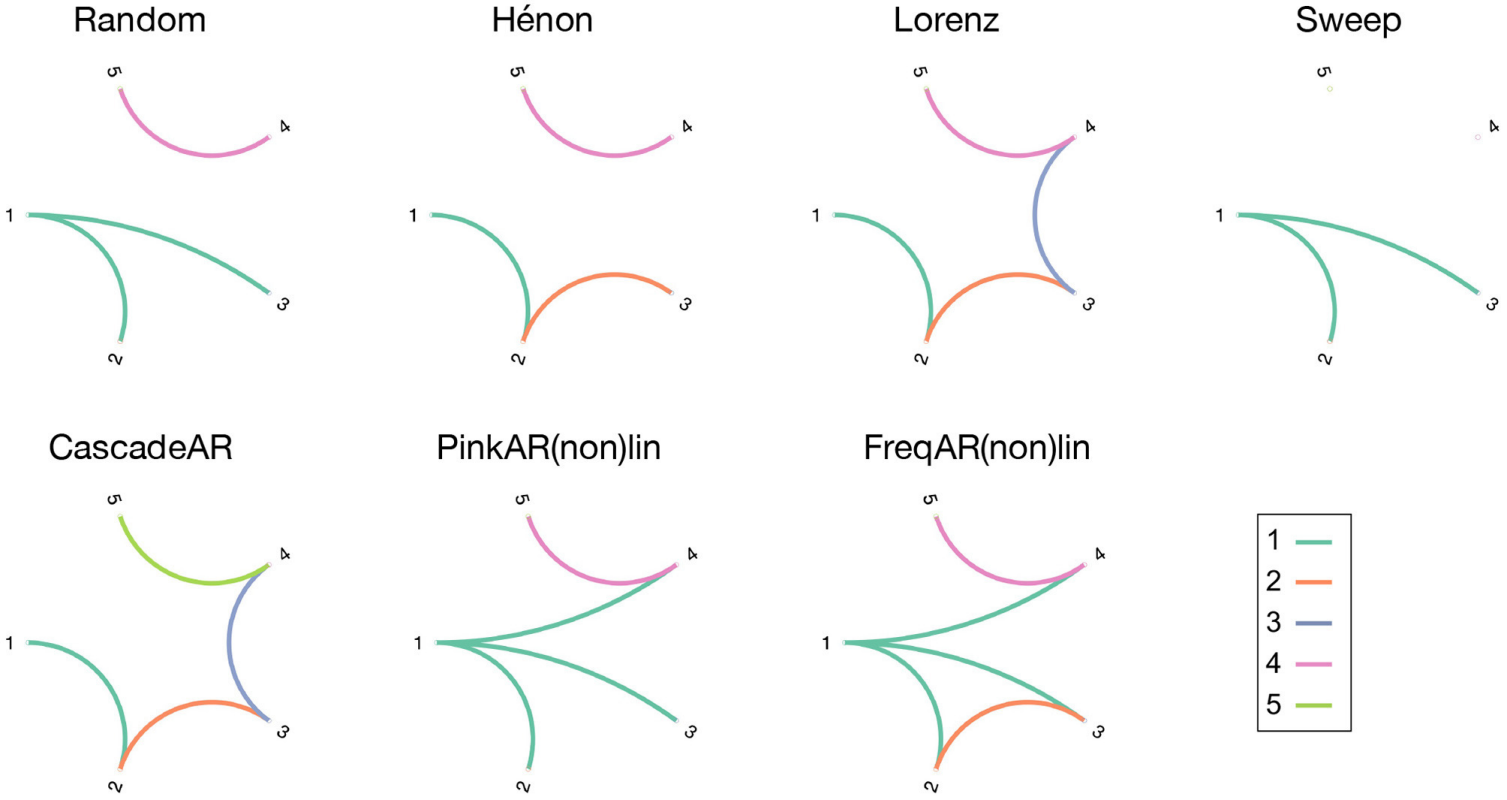
Overview figure, showing the connection diagrams of the different simulation models that were used in this study. The connections are colour-coded in function of the source channel.

To evaluate the causality measures we use a set of simulation models, which consist of several coupled and uncoupled dynamic systems. A variety of generative processes (stochastic and chaotic) and interaction types (linear, non-linear, and frequency specific) were chosen to see how well these metrics perform when applied in different contexts. This way, the generalisability of their performance in- and outside their respective “comfort zone” becomes apparent. For each model 100 realisations are simulated, on which the causality measures are computed. Figure 1 gives an overview of the simulation models' connectivity networks (see below for details).

Each simulation model consists of a dynamical system that is represented by a set of variables *X*_1_, …, *X*_*K*_. The associated *K*-variate time series are constructed by simultaneously sampling the observed variables into the set {*x*_1,*t*_, …, *x*_*K,t*_}, *t* = 1, …, *n*. All simulated signals are of length *n* = 1, 000 and sampled with *f*_*s*_ = 256 Hz. The notation *X*_2_ → *X*_1_ denotes a Granger causal relationship from *X*_2_ to *X*_1_, while *X*_2_ → *X*_1_|*Z* denotes direct Granger causality from *X*_2_ to *X*_1_, accounting for the presence of the other (confounding) variables, i.e., *Z* = {*X*_3_, …, *X*_*K*_}. A bidirectional coupling between *X*_1_ and *X*_2_ is written as *X*_1_ ↔ *X*_2_. The notation of causal relationships between other pairs of variables is analogous.

### 2.1. Coupled Randomised Signals (“Random”)

The first model is a simple stochastic system with linear interactions *X*_1_ → *X*_2_, *X*_1_ → *X*_3_, and *X*_4_ → *X*_5_.


$$\begin{array}{l} x_{1,t}=w_{1,t}\\ x_{2,t}=\left(1-c\right)w_{2,t}+c\cdot x_{1,t-3}\\ x_{3,t}=\left(1-c\right)w_{3,t}+c\cdot x_{1,t-2}\\ x_{4,t}=w_{4,t}\\ x_{5,t}=\left(1-c\right)w_{5,t}+c\cdot x_{4,t-5} \end{array}$$

*w*_*i,t*_, *i* = 1, …, 5 are drawn from independent Gaussian white noise processes with zero mean and unit variance. Coupling strength *c* = 0.5.

### 2.2. Coupled Hénon Maps (“Hénon”)

Hénon maps are one of the most studied dynamical systems that exhibit chaotic behaviour (Henon, 1976). Because the system becomes unstable for cascades of more than three coupled Hénon maps, the system is split up into two cascades of lengths 3 and 2.


$$\begin{array}{l} x_{1,t}=1.4-x_{1,t-1}^{2}+0.3x_{1,t-2}\\ x_{2,t}=1.4-cx_{1,t-1}x_{2,t-1}+0.3x_{2,t-2}\\ x_{3,t}=1.4-cx_{2,t-1}x_{3,t-1}+0.3x_{3,t-2}\\ x_{4,t}=1.4-x_{4,t-1}^{2}+0.3x_{4,t-2}\\ x_{5,t}=1.4-cx_{4,t-1}x_{5,t-1}+0.3x_{5,t-2} \end{array}$$

As the interaction terms involve multiplication of the signal values, the couplings are non-linear. Coupling strengths are again *c* = 0.5.

### 2.3. Coupled Lorenz Systems (“Lorenz”)

Originally developed as a mathematical model for atmospheric convection, Lorenz systems consist of three ordinary differential equations (Lorenz, 1963). The parameter values and initial conditions are chosen here such that the systems exhibit chaotic behaviour. Here we show the equations for the first three Lorenz systems, the 4th and 5th systems are defined similar to the 2nd and 3rd systems.


$$\begin{array}{l} {ẋ}_{1}=10\left(y_{1}-x_{1}\right)\\ {ẋ}_{2}=10\left(y_{2}-x_{2}\right)+c\left(x_{1}-x_{2}\right)\\ {ẋ}_{3}=10\left(y_{3}-x_{3}\right)+c\left(x_{2}-x_{3}\right)\\ {ẏ}_{1}=28x_{1}-y_{1}-x_{1}z_{1}\\ {ẏ}_{2}=28x_{2}-y_{2}-x_{2}z_{2}\\ {ẏ}_{3}=28x_{3}-y_{3}-x_{3}z_{3}\\ {ż}_{1}=x_{1}y_{1}-8/3z_{1}\\ {ż}_{2}=x_{2}y_{2}-8/3z_{2}\\ {ż}_{3}=x_{3}y_{3}-8/3z_{3} \end{array}$$

The coupling strength is *c* = 4. As the interactions are introduced inside the differential equations, the Lorenz systems are non-linearly coupled. To obtain the multivariate time series, we sample each system along the first dimension (i.e., *x*_*i*_, *i* = 1…5) with a sampling period of 0.5 units.

### 2.4. Seizure Model (“Sweep”)

The fourth simulation model mimics the propagation of an epileptic seizure, where seizure activity is modeled as a sine wave with time-varying frequency. The seizure starts in channel 1, and propagates to channels 2 and 3 with different delays.


$$\begin{array}{l} x_{1,t}=sin\left(2\pi f_{t}t\right)+\theta_{1,t}\\ x_{2,t}=x_{1,t-2}+\theta_{2,t}\\ x_{3,t}=x_{1,t-4}+\theta_{3,t}\\ x_{4,t}=\theta_{4,t}\\ x_{5,t}=\theta_{5,t} \end{array}$$

The frequency *f*_*t*_ starts at 12 Hz and decreases linearly until it reaches 8 Hz at the end of the signal. To mimic the signal properties of electroencephalography (EEG) signals, the time series are generated through a set of pink noise processes, θ_*i,t*_, *i* = 1, …, 5, with a 1/*f* spectral distribution. The noise amplitude is chosen such that the Signal to Noise Ratio (SNR) equals −5*dB*. This seizure model has already been applied with success in several studies to validate the performance of Granger causality-based connectivity measures (Lie and van Mierlo, 2017; van Mierlo et al., 2018).

### 2.5. Cascade AR Model (“CascadeAR”)

The remaining simulation models are created through stochastic, autoregressive processes. For the cascade AR model, all interactions are linear and arranged in such a way that the system forms a sequential cascade with bidirectional couplings in the middle (i.e., the couplings are *X*_1_ → *X*_2_, *X*_2_ ↔ *X*_3_, *X*_3_ ↔ *X*_4_, and *X*_5_ → *X*_4_).


$$\begin{array}{l} x_{1,t}=\sqrt{2}\rho x_{1,t-1}-\rho^{2}x_{1,t-2}+\theta_{1,t}\\ x_{2,t}=0.5c\left(x_{1,t-1}+x_{3,t-1}\right)+\left(1-c\right)\sqrt{2}\rho x_{2,t-1}-\rho^{2}x_{2,t-2}+\theta_{2,t}\\ x_{3,t}=0.5c\left(x_{2,t-1}+x_{4,t-1}\right)+\left(1-c\right)\sqrt{2}\rho x_{3,t-1}-\rho^{2}x_{3,t-2}+\theta_{3,t}\\ x_{4,t}=0.5c\left(x_{3,t-1}+x_{5,t-1}\right)+\left(1-c\right)\sqrt{2}\rho x_{4,t-1}-\rho^{2}x_{4,t-2}+\theta_{4,t}\\ x_{5,t}=\sqrt{2}\rho x_{5,t-1}-\rho^{2}x_{5,t-2}+\theta_{5,t} \end{array}$$

The coupling constants are *c* = 0.8 and ρ = 0.9, and θ_*i,t*_, *i* = 1, …, 5 are independent pink noise processes.

### 2.6. Pink (Non-)linear AR Models [“PinkAR(non)lin”]

This simulation model consists of a set of coupled and uncoupled AR systems, driven by independent 1/*f* processes. The following equations are true for the linear model (“PinkARlin”). For the non-linear model (“PinkARnonlin”), the interaction terms *X*_1_ → *X*_2_ and *X*_1_ → *X*_4_ are quadratic instead of linear.


$$\begin{array}{l} x_{1,t}=0.95\sqrt{2}x_{1,t-2}+\theta_{1,t}\\ x_{2,t}=0.5x_{1,t-2}+\theta_{2,t}\\ x_{3,t}=-0.4x_{1,t-3}+\theta_{3,t}\\ x_{4,t}=-0.5x_{1,t-2}+0.25\sqrt{2}x_{4,t-1}+0.25\sqrt{2}x_{5,t-1}+\theta_{4,t}\\ x_{5,t}=-0.25\sqrt{2}x_{4,t-1}+0.25\sqrt{2}x_{5,t-1}+\theta_{5,t} \end{array}$$

θ_*i,t*_, *i* = 1, …, 5 are independent pink noise processes.

### 2.7. Frequency-Dependent AR Models [“FreqAR(non)lin”]

The last simulation model also consists of a set of AR systems driven by 1/*f* processes, with interactions that are limited to specific frequency bands. The connections are limited either to the low (8–12 Hz) or high (25–100 Hz) frequencies. These frequency bands have been chosen in accordance with the α and γ bands relevant in the analysis of EEG signals. The frequency-specific connections are obtained by using band-passed versions of the 1/*f* stochastic processes in the relevant interaction terms. The following equations are true for the linear model (“FreqARlin”). For the non-linear model (“FreqARnonlin”), the interaction terms *X*_1_ → *X*_2_ and *X*_1_ → *X*_4_ are quadratic instead of linear.


$$\begin{array}{l} x_{1,t}=0.95\sqrt{2}x_{1,t-1}-0.9025x_{1,t-2}+\theta_{1,t}\\ x_{2,t}=0.5\theta_{1\gamma ,t-2}+\theta_{2,t}\\ x_{3,t}=-0.4\theta_{1\gamma ,t-3}+0.25\sqrt{2}\theta_{2\gamma ,t-3}+\theta_{3,t}\\ x_{4,t}=-0.5x_{1\alpha ,t-5}+0.25\sqrt{2}x_{4,t-1}+0.25\sqrt{2}x_{5,t-1}+\theta_{4,t}\\ x_{5,t}=-0.25\sqrt{2}\theta_{4\gamma ,t-1}+0.25\sqrt{2}x_{5,t-1}+\theta_{5,t} \end{array}$$

θ_*i,t*_, *i* = 1, …, 5 are independent 1/f pink noise processes. The model is constructed such that the system is separated into two fictitious brain regions with high-frequency intra-regional (*X*_1_ → *X*_2_, *X*_2_ → *X*_3_ and *X*_4_ → *X*_5_ in the γ band) and low-frequency inter-regional connections (*X*_1_ → *X*_4_ in the α band), concordant with the current hypothesis of the different roles of these frequency bands in communication within the brain. The connections are restricted to their respective frequency bands by using a bandpass-filtered version of the respective sources (θ_*iα,t*_ and θ_*iγ,t*_).

## 3. Causality Measures

In this section we give a short explanation of the causality measures used in this study, which were selected based on their popularity in literature. It should be noted that most causality measures require the input signals to be stationary (i.e., the mean and variance don't change in time). If this is not the case, the signals must always be pre-processed to make them stationary, e.g., by working with one of their derivatives (if these are stationary) or by using pre-whitening algorithms.

### 3.1. Cross-Correlation

Cross-correlation is a statistical concept that estimates the linear interdependence between two signals as a function of the time shift of one relative to the other. Its value depends on this time shift, τ, and is formulated as follows:


(1) $$\begin{array}{r} {\hat{\rho }}_{x,y}\left(\tau \right)=\frac{1}{N-\tau }\overset{N-\tau }{\underset{n=1}{\sum }}\frac{\left(x_{n}-\bar{x}\right)\left(y_{n+\tau }-ȳ\right)}{\sigma_{x}\sigma_{y}} \end{array}$$

Resulting values range between −1 and 1 and indicate the strength of the linear relation between the two variables. The cross-correlation is zero in absence of a linear relationship between the variables, and reaches 1 or −1 in case of perfect correlation or anti-correlation, respectively. By looking for the time shift where the magnitude of the cross-correlation is maximal, this technique can also be used to estimate the delay between observed signals (see section 4). Cross-correlation is a bivariate measure, but can easily be applied to multivariate systems by repeating the analysis for all possible signal pairs. However, when two variables are driven by a common source a spurious connection may be found between the first two, even though manipulation of one of these variables won't necessarily influence the values of the other. Cross-correlation is thus unable to differentiate relations caused by latent confounding variables. Moreover, correlation only detects linear relationships. This is the least advanced metric used in this study and will probably be unable to reliably detect the connections in all simulation models. Cross-correlation is included here because it is still a widely used measure, and will act as a baseline against which performance of the other measures can be compared. Before computing the cross-correlation the data is pre-whitened with a non-parametric approach based on the Singular Value Decomposition (SVD) of the covariance matrix of the time-series (Hansen and Jensen, 2005).

### 3.2. Granger Causality Index

Many causality measures are based on the concept of Granger causality, which investigates whether observations of one signal can be used to predict another (Seth et al., 2015). Y is said to Granger-cause X if it can be shown that the past values of both X and Y give a better prediction of X, compared to predictions based on the past values of X alone. The predictions are based on autoregressive (AR) modelling, where signals are decomposed into a linear combination of their past values plus additional uncorrelated white noise. Multivariate AR (MVAR) can be expressed as:


(2) $$\begin{array}{r} \text{X}\left(n\right)=\overset{p}{\underset{m=1}{\sum }}\text{A}\left(m\right)\text{X}\left(n-m\right)+\text{E}\left(n\right) \end{array}$$

Here, $\text{X}\left(n\right)={\left[x_{1}\left(n\right)x_{2}\left(n\right)\ldots x_{K}\left(n\right)\right]}^{T}$ is the signal matrix at time point n, $\text{E}\left(n\right)={\left[e_{1}\left(n\right)e_{2}\left(n\right)\ldots e_{K}\left(n\right)\right]}^{T}$ is the noise matrix at time point n, *p* is the model order and **A**(*m*) is the *K* × *K* coefficient matrix for delay m. The model order determines the number of past values included in the predictions. The optimal model order can be determined automatically using several metrics. In this study we use Schwarz's Bayesian Criterion (Schwarz, 1978).

Bivariate Granger causality from Y to X can be derived by comparing the residuals of X obtained by fitting a univariate AR model and a bivariate model of X and Y. The Granger Causality Index (GCI) is computed using the following expression:


(3) $$\begin{array}{r} GCI_{xy}=ln\left(\frac{V_{x|x}}{V_{x|xy}}\right) \end{array}$$

where *V*_*x*|*x*_ is the variance of the residuals in the univariate case, and *V*_*x*|*xy*_ is the residual variance for the bivariate model. If the past values of Y don't improve the predictions of X, then *V*_*x*|*x*_ ≈ *V*_*x*|*xy*_ and hence *G*_*xy*_ ≈ 0 (Granger, 1969). Improved predictions will reduce the bivariate variance *V*_*x*|*xy*_, which results in a *GCI*_*xy*_ larger than zero. The larger the improvement of the prediction, i.e., the more influence Y has on X, the larger the GCI value. Because of the linear characteristics inherent to AR modelling, the application of GCI is limited to the detection of linear connections. It should also be noted that AR models require the input signals to be stationary to obtain reliable results.

### 3.3. Conditional Granger Causality Index

Since GCI is a bivariate measure, it is incapable of dealing with latent confounding variables and often leads to false positives. In order to partly mediate this problem, we can apply its multivariate extension called the Conditional Granger Causality Index (CGCI). By considering all K variables at the same time, it can account for possible effects caused by common drivers in the rest of the system (Z). The effect of Y on X is now calculated by looking at the residuals of a VAR model with all K variables and a VAR model of all variables except for Y.


(4) $$\begin{array}{r} CGCI_{Y\to X|Z}=ln\left(\frac{V_{x|xz}}{V_{x|xyz}}\right) \end{array}$$

where *V*_*x*|*xz*_ is the variance of the restricted model (all variables except Y) and *V*_*x*|*xyz*_ of the unrestricted model (all K variables).

### 3.4. Partial Directed Coherence

Using Fourier methods the AR model can be decomposed into its frequency domain representation, which allows for spectral causality analysis. Intuitively, these measures quantify the fraction of the spectral power, at a given frequency *f*, of the driver *X* that contributes to the future of the response variable *Y*. Analysing the connectivity in the frequency domain offers several advantages when working in fields where frequency-specific connections and modulations are assumed, such as in neuroscience applications. The Fourier transformation of the AR model is calculated as:


$$\begin{array}{l} \text{E}\left(f\right)=\text{A}\left(f\right)\text{X}\left(f\right) \end{array}$$

where


(5) $$\begin{array}{r} \text{A}\left(f\right)=-\overset{p}{\underset{m=0}{\sum }}\text{A}\left(m\right)e^{-i2\pi \frac{f}{f_{s}}m} \end{array}$$

with *f*_*s*_ the sampling frequency and **A**(0) = −**I** the negative identity matrix. **E**(*f*), **A**(*f*) and **X**(*f*) are the Fourier transform of the white noise, AR coefficient and time series matrices, respectively. The Partial Directed Coherence (PDC) was defined by Baccala and Sameshima with the following equation (Baccalá and Sameshima, 2001):


(6) $$PDC_{ij}(f)=\frac{|A_{ij}(f){|}^{2}}{\sum_{k=1}^{K}{\left|A_{kj}(f)\right|}^{2}}$$

*PDC*_*ij*_(*f*) values lie within the interval [0,1] and give, for each frequency *f*, the ratio of the information transfer from channel j to i, normalised w.r.t. the total outflow from channel j. Because of the normalisation to channel outflow, unidirectional flows are enhanced compared to multiple flows of the same intensity that leave from a channel. PDC therefore emphasises on sinks, rather than sources. PDC detects only direct information flow, contrary to the Directed Transfer Function (DTF), which also detects indirect information flows (see below).

### 3.5. Directed Transfer Function

Directed Transfer Function (DTF) is, next to PDC, a second widely used spectral causality measure. The computation starts from the transfer matrix **H**(*f*), which is the inverse of the Fourier transformed MVAR coefficient matrix:


$$\begin{array}{l} \text{X}\left(f\right)={\text{A}}^{-1}\left(f\right)\text{E}\left(f\right)=\text{H}\left(f\right)\text{E}\left(f\right) \end{array}$$

The DTF is expressed through the equation (Kaminski and Blinowska, 2014):


(7) $$DTF_{ij}(f)=\frac{|H_{ij}(f){|}^{2}}{\sum_{k=1}^{K}{\left|H_{ik}(f)\right|}^{2}}$$

*DTF*_*ij*_ is normalised w.r.t. the total incoming information flow toward channel i, and therefore emphasises sources rather than sinks. As mentioned earlier, DTF detects direct as well as indirect connections. In some cases this can be useful, e.g., to find the driving node of a network (i.e., the one that has the most influence, direct or indirect, on the other nodes in the network). This has been used with success for example to identify the seizure onset zone in patients with epilepsy (Staljanssens et al., 2017). Both PDC and DTF are computed within the frequency range *f* = [1 − 30]*Hz*, except in the clinical application. Here a frequency range *f* = [3 − 30]*Hz* is used (see section 5).

### 3.6. Partial Mutual Information on Mixed Embedding

All previous methods fit a mathematical model to the observed time series by minimising an optimisation function (e.g., least means squares error). However, in practice we rarely know the generative processes behind the observed signals and they may not be well-represented by the assumed model. This results in bad model fits and often false connections that are returned with high confidence. Because of these problems, several measures coming from information theory are gaining in interest. Information theory provides a natural framework for non-parametric methods to detect statistical relationships between signals. These methods can therefore be applied to all kinds of generative processes and interaction types, also for systems with non-linear coupling dynamics (which can't be detected with any of the previous methods).

Information theoretic measures are based on the concept of Mutual Information (MI), which quantifies the amount of information that is gained about a variable by observing a second variable. Partial Mutual Information on a Mixed Embedding (PMIME) uses this principle to construct a mixed embedding vector that best explains the future of the response variable *X*_1_. At each iteration, conditional mutual information (CMI) is used to find the most informative (lagged) variable which is then added to the embedding vector. If the amount of added information is too little, the embedding procedure is stopped. This way we obtain a mixed embedding that contains various delays of all K variables *X*_1_, …, *X*_*K*_, that best explain the future of the response variable *X*_1_, defined as ${\text{x}}_{1,t}^{h}=\left[x_{1,t+1},\ldots ,x_{1,t+h}\right]$. The embedding vector, **w**_*t*_, can be decomposed into the subsets ${\text{w}}_{t}^{X_{1}}$, ${\text{w}}_{t}^{X_{2}}$ and ${\text{w}}_{t}^{Z}$ which contain the lagged components of the response variable *X*_1_, driving variable *X*_2_ and confounding variables in *Z*, respectively. The PMIME metric is then defined as:


(8) $$\begin{array}{r} PMIME_{X_{2}\to X_{1}|Z}=\frac{I\left({\text{x}}_{1,t}^{h};{\text{w}}_{t}^{X_{2}}|{\text{w}}_{t}^{X_{1}},{\text{w}}_{t}^{Z}\right)}{I\left({\text{x}}_{1,t}^{h};{\text{w}}_{t}\right)} \end{array}$$

PMIME can be considered a normalised version of the Partial Transfer Entropy (PTE) for optimised non-uniform embeddings of all K variables. The values of PMIME range between zero and one, where zero indicates the absence of components from *X*_2_ in the mixed embedding vector and, consequently, no direct Granger causal relationship from *X*_2_ to *X*_1_. The search space for the mixed embedding vector is limited by a maximum lag, *L*_*max*_. This parameter can be set to an arbitrarily large number without affecting the performance, but the computational cost increases dramatically due to the many evaluations of CMI between the lagged variables. Here we use *L*_*max*_ = 5, for more information on this measure we refer the reader to the original paper by Kugiumtzis (2013). The *k*-nearest neighbours method is used for estimation of MI and CMI.

### 3.7. Surrogate Testing: Phase Shuffling

Each causality measure returns a value that quantifies the strength of the detected relationship between the observed variables. To evaluate which values are significant, a suitable threshold must be found that allows to distinguish between non-zero outputs caused by noise or genuine interactions. Based on a comparative study of different statistical tests (see Supplementary Data Sheet 1), we perform this test with the help of phase-shuffled surrogate data. In this study we provide a comparative evaluation of the outcome of the connectivity analysis when combined with parametric tests, block permutation surrogates or phase shuffling surrogates. The use of phase shuffling surrogates showed the best performance overall, with better robustness to conditions that deviate from the underlying assumptions compared to the other tests. The aim is to create a large set of artificial time series, based on the original signals, wherein any possible causal influence between the variables is destroyed whilst preserving as much as possible the other signal characteristics. By re-computing the causality measures on this surrogate dataset, we obtain a null distribution to which the original connectivity value can be compared.

Generating surrogate data with phase shuffling involves, as the name suggests, shuffling the signal phases. Because phase changes in the frequency domain are associated with temporal translations in time domain (and vice versa), changing the phase of a signal has a similar effect as creating time-shifted surrogate data and can effectively destroy temporal relationships between time series. Compared to sample shuffling, phase shuffling has the advantage of preserving the spectral distribution of the original signal and hence creates surrogate time series with dynamics that better approach the original signals. The phase shuffled surrogate data is created by transforming the signal to the frequency domain using the discrete Fourier transform, and then assigning random phase values (taken uniformly from the range [0, 2π]) to each spectral component. With the inverse Fourier transform the signal is brought back to the time domain and the surrogate time series are obtained. If the surrogate data needs to be a real signal (i.e., not complex), one should take care that the phases are made anti-symmetric before applying the inverse Fourier transform.

Since the output values are assumed to be zero for all measures in the absence of causality, a one-sided rank test can be used to evaluate the significance of the original causality measure value. If *r* is the rank of the original estimate of the causality measure, the *p*-value is *p* = 1 − *r*/(*n*_*surr*_ + 1), with *n*_*surr*_ = 100 the number of surrogate datasets. Connectivity values are deemed statistically significant if the *p*-value is smaller than 0.05.

### 3.8. Evaluation Metric: Matthew's Correlation Coefficient

Evaluation of the causality measures is based on their ability to detect causal effects between each possible pair of variables, and at the same time robustly reject spurious connections in the simulated systems. To quantify the measures' performance we use Matthew's correlation coefficient (MCC), a robust measure of the quality of binary (two-class) classification algorithms. MCC is regarded more informative than other measures, such as accuracy, sensitivity, specificity, and F1 score, as it takes into account the balance ratios of all four confusion matrix categories [i.e., true positives (TP), true negatives (TN), false positives (FP) and false negatives (FN)]. The MCC is equal to the correlation between the predictions and the truth, and its values range between −1 and 1. and is computed as follows:


(9) $$\begin{array}{r} MCC=\frac{\left(TP\cdot TN\right)-\left(FP\cdot FN\right)}{\sqrt{\left(TP+FP\right)\left(TP+FN\right)\left(TN+FP\right)\left(TN+FN\right)}} \end{array}$$

## 4. Lag Estimation

The dynamics of a network are governed by connections between different network nodes that allow them to communicate with and influence each other. This communication never happens instantaneously and time delays are therefore an integral part of all real-world networks. Variations in this delay (or lag) between communicating variables can lead to large changes in the dynamics of the entire system (Jirsa, 2008). However, while a large effort has already been put in trying to retrieve the correct connectivity pattern, estimating the lag between the variables has been largely overlooked. In this paper, we evaluate four methods for estimating the lags in multivariate time series, based on cross-correlation, autoregressive modelling, and information theory.

### 4.1. Cross-Correlation

Inferring connectivity from cross-correlation involves finding the time shift for which the strength of correlation between two observed signals becomes maximal. The lag value for which this maximum is reached can therefore be used as an estimator of the interaction delay between the time series.


(10) $$\begin{array}{r} \tau_{delay}=\underset{\tau \in \mathbb{Z}}{arg\;max}\left({\hat{\rho }}_{x,y}\left(\tau \right)\right) \end{array}$$

where ${\hat{\rho }}_{x,y}\left(\tau \right)$ is the cross-correlation of time-series *x* and *y*, evaluated at time-shift τ. Just like the connectivity estimate, lag estimations through cross-correlation are subject to the same limitations caused by the underlying linear assumptions (see section 3.1).

### 4.2. Autoregressive Model

In this section we introduce two new methods for estimating the lag between time series, based on autoregressive modelling. AR models allow for more robust detection of linear relationships between multivariate time series and are therefore expected to outperform cross-correlation in estimating the lags between communicating variables from multivariate systems. We follow two approaches, one in the time domain and one in the frequency domain.

#### 4.2.1. Time Domain

Looking at Equation (2), the matrix **A**(*m*) contains the AR coefficients for delay *m*. For model order *p*, each signal *X*_*i*_, *i* = 1, …, *K* of a K-variate system is modelled by the following equation:


$$\begin{array}{l} X_{i}\left(n\right)=\overset{K}{\underset{j=1}{\sum }}\overset{p}{\underset{m=1}{\sum }}\text{A}\left(i,j,m\right)X_{j}\left(n-m\right) \end{array}$$

The influence of the lagged values of *X*_*j*_ on the response variable *X*_*i*_ is thus expressed through the coefficients in **A**(*i, j, m*), *m* = 1, …, *p*. If a lagged component has no predictive value its coefficient will be close to zero, whereas strongly predictive components will lead to coefficients with a large magnitude. Similar to the cross-correlation method, we can thus identify the lagged component of the variable *X*_*j*_ that is most predictive for *X*_*i*_ using the argmax function:


(11) $$\begin{array}{r} \tau_{delay}\left(i,j\right)=\underset{m=1,\ldots ,p}{arg\;max}\left(\left|A\left(j,i,m\right)\right|\right) \end{array}$$

The AR-based lag estimation in time domain involves finding the maximal values along the third dimension of the *K* × *K* × *p* coefficient matrix **A**, that's constructed by appending the AR coefficient matrices to each other in order of ascending delay. The relative magnitude of the lagged components is more or less independent on the model order, p, which should thus be chosen large enough to incorporate all possible interaction delays. In this study we use a fixed model order *p* = 20 for the AR(p)-based lag estimations.

#### 4.2.2. Frequency Domain

By again making use of the interplay between phases in frequency domain and shifts in time domain, we can estimate the delay between communicating variables by looking at the phases of the AR model. From Equation (5), the spectral components of the Fourier-transformed coefficient matrix **A**(*f*) are formed by a summation of the AR coefficient matrices for each delay, weighted with an exponential function $e^{-i2\pi \frac{f}{f_{s}}m}$. Each spectral component can thus be decomposed into a sum of vectors with magnitude **A**(*m*) and phase −2π(*f*/*f*_*s*_)*m*. If a specific component in **A**(*m*) is significantly larger than the other values, its associated phase will dominate the phase of the spectral components.

The most prominent phase value across all considered frequencies is thus an indicator of a large component in **A**(*m*). To estimate which phase predominates in the spectral components, we use a kernel density estimation on the phase values of the coefficients in each array **A**(*i, j, f*), with *f* ranging over all considered frequency values and (*i, j*) fixed depending on the considered driving and response variables (*X*_*j*_ and *X*_*i*_, respectively). Kernel density estimation is a non-parametric method to make inferences on the probability density function, based on a finite data sample. It uses a linear combination of time-shifted kernels to estimate the true underlying density function and can be seen as a smooth and continuous alternative for histograms. The phase value at the maximum peak of the density function, ϕ_*max*_, is then transformed to the most predictive lag component as:


(12) $$\begin{array}{r} \tau_{delay}=\frac{\phi_{max}}{2\pi }\frac{f_{s}}{f} \end{array}$$

### 4.3. Partial Mutual Information on Mixed Embedding

PMIME lends itself naturally to estimate the lag between variables, as the method consists of creating a mixed embedding vector by iteratively adding the lagged component that holds the most information on the future of the response variable *X*_*i*_. If we want to estimate the lag of the effects coming from driving variable *X*_*j*_, we simply look at the lagged component of *X*_*j*_ that was added first to the embedding vector. The non-parametric nature of PMIME should allow this method to also robustly estimate the lag between variables with non-linear interactions.

### 4.4. Simulation Models for Evaluating the Lag Metrics

To evaluate the performance of the proposed lag estimation methods, we designed slightly altered versions of the “Sweep,” “FreqARlin,” and “PinkAR(non)lin” simulation models, where we introduce a variable lag between two communicating variables. This way, we can track whether the lag estimation metrics are able to detect the correct lag between communicating variables.

The first system consists of a bivariate compression of the “Sweep” model, where only the variables *X*_1_ and *X*_3_ are retained. With this system we can evaluate how the metrics perform on bivariate systems with periodic signals. Then we move to signals of an autoregressive nature by extracting the *X*_1_ and *X*_3_ variables from the “PinkARlin” system. In the third system, we apply the lag estimation methods on the entire multivariate “PinkARlin” model, where again the lag for the connection *X*_1_ → *X*_3_ is made variable. We also check how the metrics behave for non-linear interactions by doing the same analysis as with the previous system, but applied on the “PinkARnonlin” simulation model (where the connection *X*_1_ → *X*_3_ is quadratic, see section 2). Lastly we have a model with frequency-specific connections, where we start from the “FreqARlin” system and made the lag between *X*_1_ and *X*_2_ variable. Because changes in the lags can introduce instabilities in chaotic simulation models, these were not included in this analysis. Each system is realised 100 times for each lag value, ranging from 1 to 20 sample points.

## 5. Application on sEEG Seizure Data

**Figure 2:**
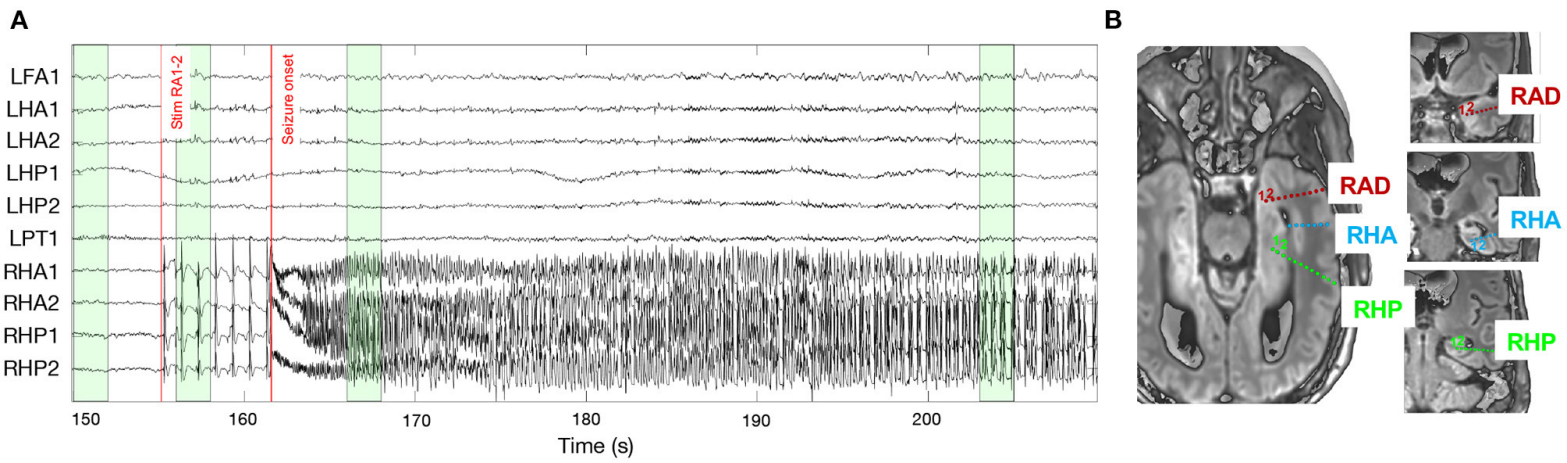
**(A)** Local field potentials as measured from the contacts included in the connectivity analysis. The two red lines indicate the start of the stimulation in the right amygdala, and the onset of the epileptic seizure. The green boxes highlight the epochs on which connectivity is computed (*t* = 150 − 152, 156 − 158, 166 − 168, and 203 − 205 s). The epoch at the end of the EEG recording is not visualised because it lies far outside the displayed window (*t* = 985 − 990 s). **(B)** Position of the implanted electrodes in the right amygdala (RAD, red), anterior hippocampus (RHA, blue), and posterior hippocampus (RHP, green). Because of the implantation angle of the electrodes, some contacts are not visible on the axial plane (e.g., for the right anterior hippocampus, the most medial contacts cannot be seen), so coronal views (right) are also added. On each electrode, the most medial contact corresponds to contact 1.

To validate the conclusions of the simulation study, the same methods for connectivity and lag estimation are applied to a stereo-electroencephalogram^1^ (sEEG) that contains ictal activity. The seizure was recorded during a clinical protocol where trains of repetitive electrical stimulations were applied to contact pairs in order to map cortical functions and excitability. The entire sEEG setup consists of depth electrodes in the frontal lobe, posterior cingulate, parietal lobe, and hippocampus, both on in the left and right hemisphere. Upon stimulating the most medial contacts of the right amygdala (RA1-2), a focal seizure was elicited in the right mesio-temporal brain region. Figure 2 shows the intracranial EEG measured from a selection of contacts, together with the position of the electrodes in the right hemisphere. Two red bars indicate the start of stimulation and of the seizure, which can be identified as fast, high-frequency activity in some of the contacts in the right anterior and posterior hippocampus (RHA and RHP, respectively). The signals from the RA1-2 contacts cannot be interpreted because they are riddled with artefactual activity due to the stimulation current blocks.

The connectivity and lag values are estimated by applying the aforementioned metrics on four epochs of 2s (*f*_*s*_ = 1 kHz) from the sEEG signals before stimulation (i.e., resting state, *t* = 150 − 152 s), during stimulation but before the seizure (*t* = 156 − 158 s), at the beginning of the seizure (*t* = 166 − 168 s), near the end of the seizure (*t* = 203 − 205 s), and at the end of the sEEG recording when no seizure activity and stimulation artifacts are present (*t* = 985 − 987 s). The sEEG signal and electrode locations can be seen in Figure 2. Because sEEG signals have a more complex structure than the simulation models, the surrogate data, used to detect only significant interactions, is generated using the iterative amplitude adjusted Fourier transform (iAAFT) method. This is an improved version of the phase shuffling method that was used for the simulation models, and preserves the autocorrelation and amplitude distribution of the signals (Schreiber and Schmitz, 1996). For the simulation models the phase shuffling method is still used to reduce the computational cost and because the simple data structure makes the added value of iAAFT redundant and without any impact on the results. The frequency-dependent causality measures (PDC and DTF) are computed within the frequency band *f* = [3 − 30] Hz. The variability of the lag estimations is also investigated by computing the lag metrics on a sliding window of 2s throughout the entire seizure (*t* = 168 − 210 s). The included channels are: the first two contacts in the right anterior and posterior hippocampus (RHA/RHP1-2), where the seizure activity is most prominently present. The first contact in the left parietal and frontal anterior regions (LPT/LFA), which were unaffected by the seizure. The first two contacts in the left anterior and posterior hippocampus (LHA/LHP1-2) to inspect the hippocampal network during the seizure. The stimulated contacts (RA1-2) are not included in the analysis because they are polluted with stimulation artifacts which would skew the results.

## 6. Results

### 6.1. Causality Measures

**Figure 3:**
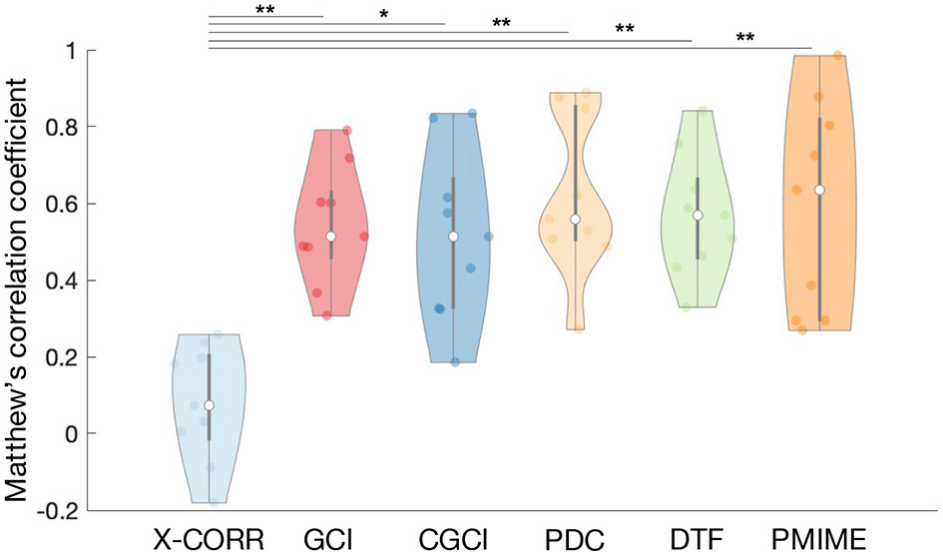
Violin plot of the MCC values for the different causality measures. The white dots and black lines indicate the median and quartile values. The width of the violin illustrates the density of the MCC distribution. Statistically significant differences (one-sided *T*-test) are annotated with a black line and star (*p < 0.05, **p < 0.005).

Here we evaluate all selected causality measures and inspect how their performance changes when they are computed over the different simulation models. All connections are tested for statistical significance based on the phase shuffling test, except for PMIME where all non-zero values are statistically significant by design (see Supplementary Data Sheet 1 for more details). Figure 3 shows the distribution of the MCC values for each causality measure using a violin plot.^2^ We found that all causality measures had similar results with overlapping distributions except for a clearly lower performance of cross-correlation. A one-sided *T*-test confirmed that the MCC values of cross-correlation are significantly lower than those of the other causality measures (significance level α = 0.05). This trend was also observed during the evaluation of the statistical tests (see Supplementary Data Sheet 1). On average PMIME shows the best performance, but the difference with the other measures is small and the MCC distributions largely overlap.

**Figure 4:**
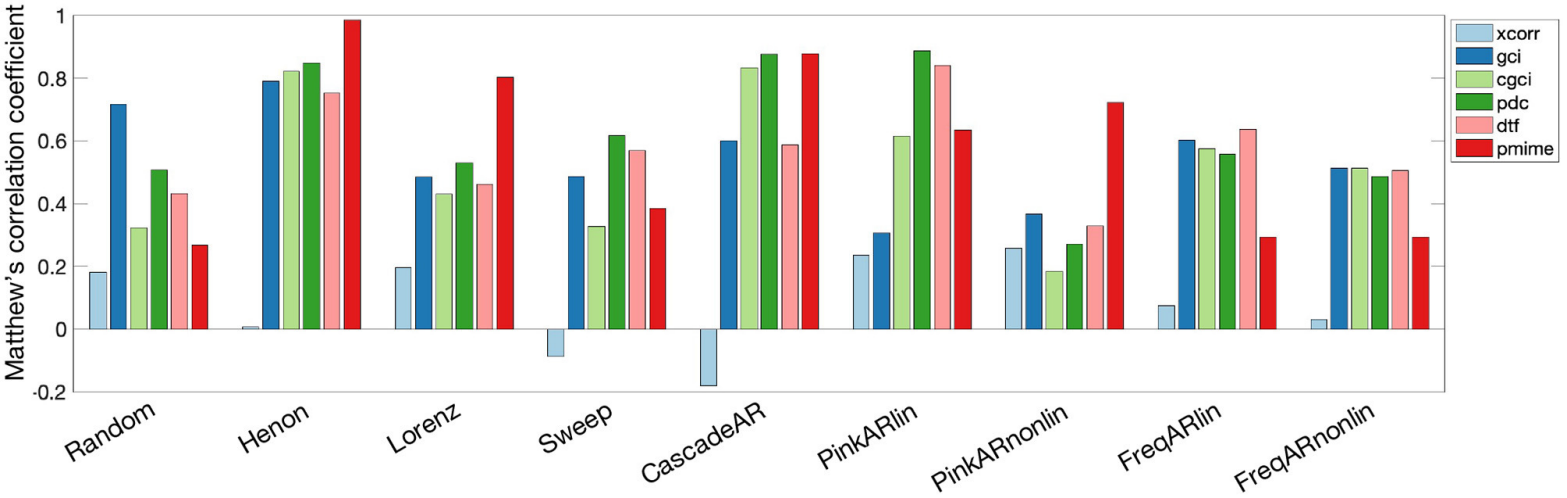
Bar graph showing the performance of the causality measures on each simulation model separately. The measures are computed on 100 iterations of each simulation model and performance is quantified by the MCC values.

The measures' performance for the different simulation models is shown in Figure 4. A large difference in performance can be seen for all measures between the simulation models “Random” and “Henon.” PMIME gives the best performance for the chaotic models (“Henon” and “Lorenz”) and the non-linear AR model “PinkARnonlin,” but doesn't do well on the systems with frequency-specific connections. For the latter systems and linear AR models, PDC and DTF show the best results. The exact MCC values as well as the sensitivity and precision values are summarised in Supplementary Tables 1, 2, respectively. In general we observe a higher sensitivity compared to the specificity (i.e., more false positives than false negatives). We also did a sensitivity analysis to further investigate the relationship between some of the model parameters (signal length *n* and AR model order *p*) on the outcome of the causality measures. The resulting MCC values can be seen in Supplementary Table 3. The results show a general trend of increased performance for longer signal length and higher AR model order.

**Figure 5:**
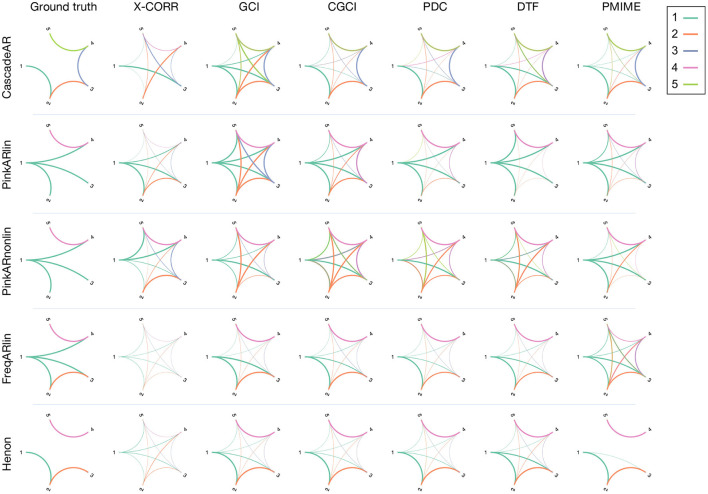
Connectograms estimated by the different causality measure for a selection of four simulation models. The connections are colour-coded in function of the source channel and the width is proportional to the number of significant interactions found over 100 realisations of the simulation model. The first column contains the ground truth network.

A detailed analysis of the average reconstructed networks over the 100 realisations of the simulation models is shown in Figure 5 for the most illustrative systems: “CascadeAR,” “PinkARlin,” “PinkARnonlin,” and “FreqARlin.”

Looking at the reconstructed connectograms for the “CascadeAR” model, many (false) indirect links are returned by GCI and to a lesser extent also by DTF (specificity 52 and 77%, respectively, see Supplementary Table 1). Better results are obtained with CGCI, PDC, and PMIME which compute networks that closely resemble the ground truth. Similar observations can be made for the results of the “Henon” model, but here the difference between GCI and CGCI is less pronounced. For this model, only PMIME is able to separate the two groups of connected signals, and returns an almost perfectly reconstruction of the true network topology.

For “PinkARlin” the network returned by PDC corresponds most to the ground truth. Both DTF and PMIME have a high probability of returning the indirect connection *X*_1_ → *X*_5_.

When non-linear interactions are introduced, such as in “PinkARnonlin,” we see that only PMIME retains its good performance and returns a network similar to the ground truth. PDC and DTF on the other hand now return a large amount of spurious connections.

In “FreqARlin” we see a relatively good performance from GCI, CGCI, PDC, and DTF, except that none of the measures is able to detect the low-frequency interaction *X*_1_ → *X*_4_. In the Supplementary Materials, an overview figure is included with the connectograms for every combination of causality measure and simulation model (see Supplementary Figure 1).

### 6.2. Lag Estimation

**Figure 6:**
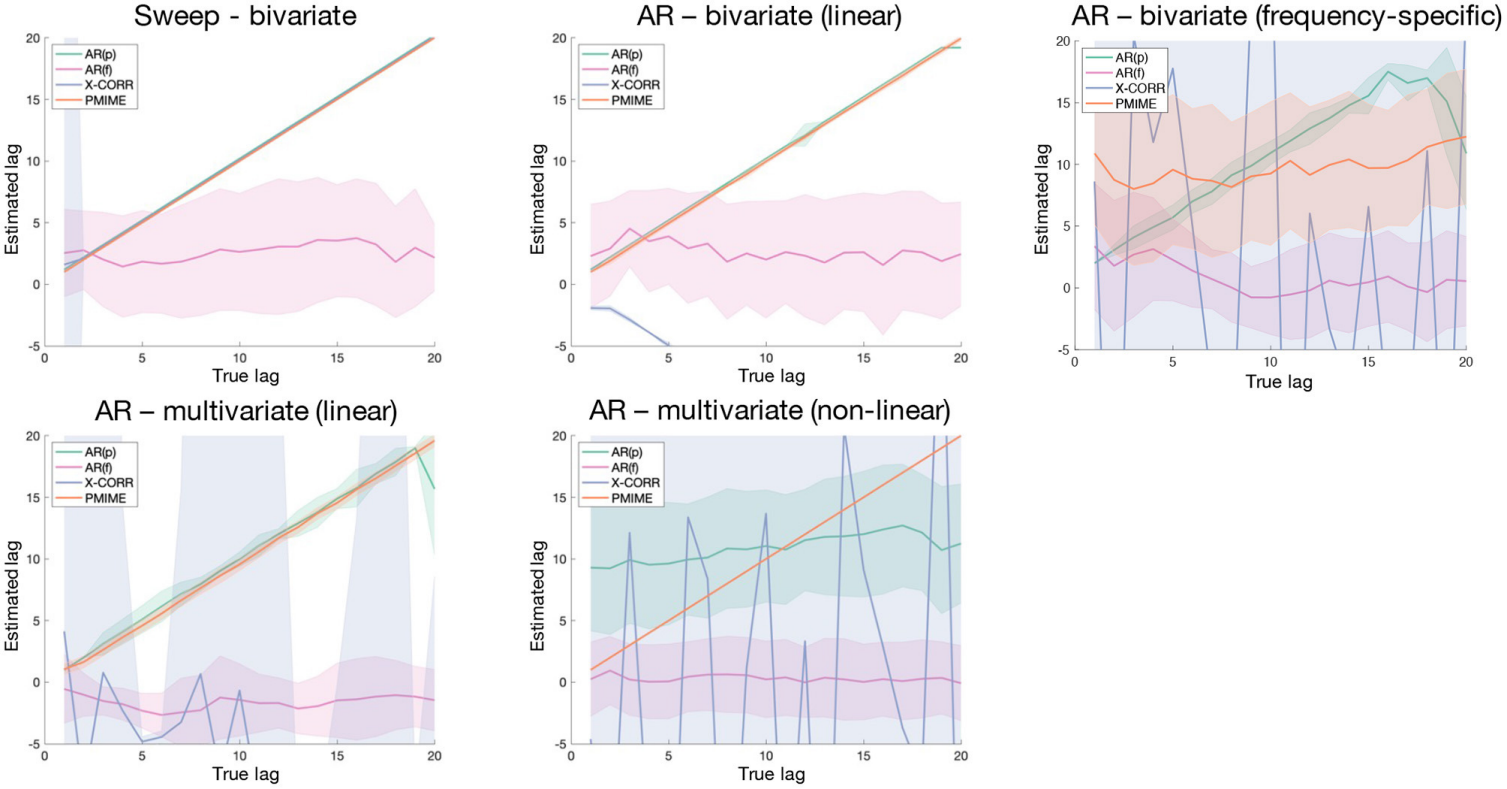
Shaded error bar plots showing the lag estimation in function of the true underlying lag values. The shaded area shows the standard deviation of the estimated lag over 100 realisations of the simulation model.

Figure 6 shows the results of the simulation models with variable lags, with the estimated lags plotted in function of the true underlying lag. At first glance we can immediately observe a poor performance of the AR(f) lag estimation metric, which always has an estimated lag value distribution centred around zero. Cross-correlation works well for the bivariate periodic (Sweep) and AR models, apart from a small mishap in the AR model where the negative lag is returned due to an incorrectly assigned directionality. In the other models cross-correlation is unable to detect the correct lag. The time-domain AR metric [AR(p)] is able to assign the correct lag value in all models with linear connections, but not in the non-linear one. In the frequency-specific and multivariate linear AR models a deviation from the true lag values is seen as we approach the model order (*p* = 20). Lag estimations based on PMIME work perfectly for all models, except for the one with frequency-specific connections.

### 6.3. Application on sEEG Seizure Data

**Figure 7:**
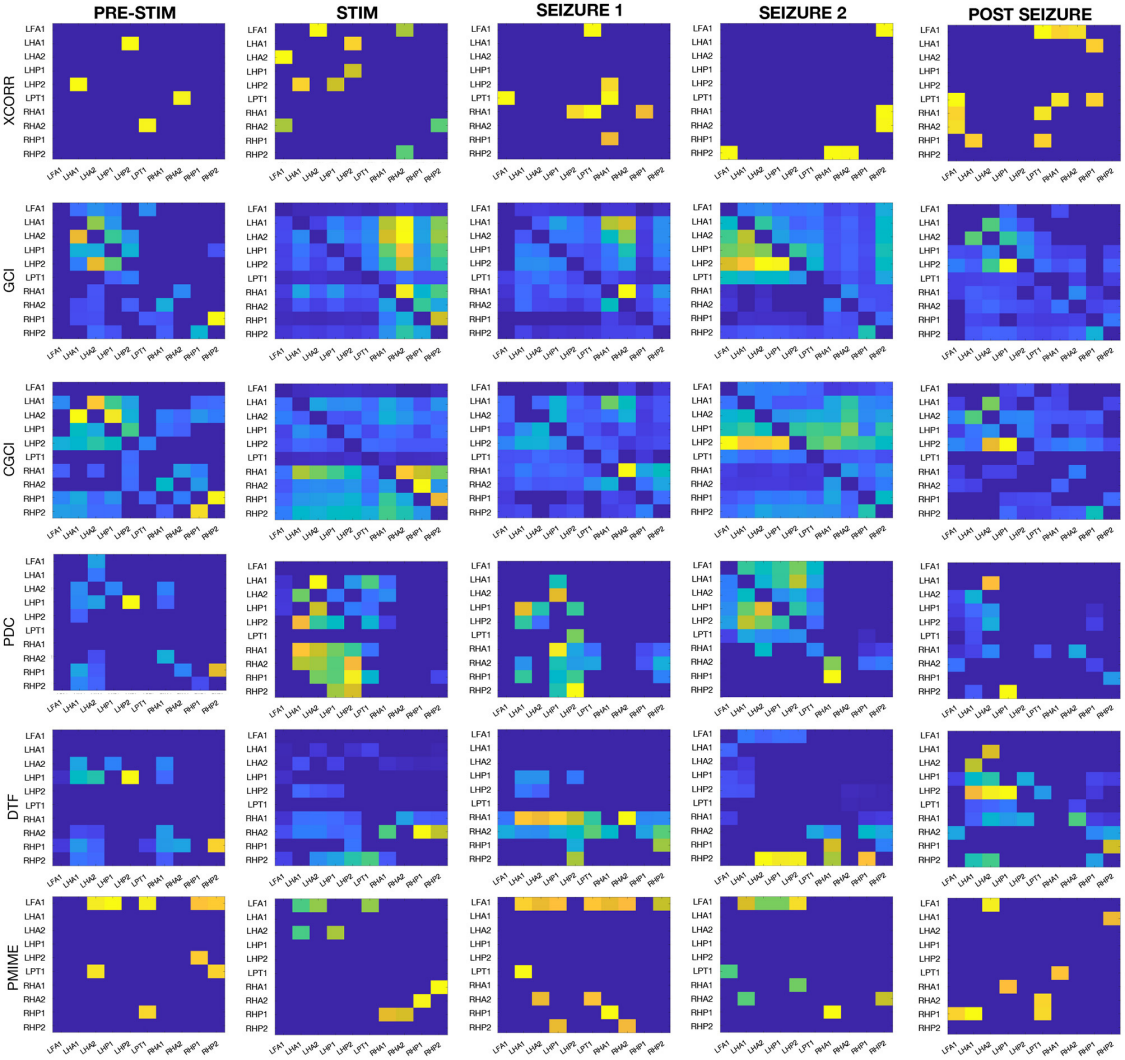
Connectivity results from the sEEG data. Each row contains the connectivity matrices from one of the causality metrics when applied on each of the epochs before/during stimulation, at the beginning/end of the seizure, and after the seizure.

The same connectivity and lag estimation methods are applied to a sEEG dataset containing a seizure triggered by electrical stimulation. Figure 7 shows the seizure networks, as reconstructed by the different connectivity metrics for all four sEEG epochs. In the connectivity matrices, each element (*i, j*) represents the strength of the connection from signal *i* toward signal *j*, taking into account only the interactions that were indicated as statistically significant after surrogate testing with iAAFT.

If we look at all AR-based measures together (GCI and CGCI in time domain, and PDC and DTF in frequency domain), a general trend can be spotted in the network behaviour during the different epochs. Pre-stimulation the connections are mostly intra-regional, with a strong cluster in the left hippocampus and to lesser extent also in the right counterpart. During stimulation the inter-hippocampal connections become stronger, mostly from the right to the left hippocampus. At the beginning of the seizure the connectivity pattern shows low overall connectivity and no clear clustering of contacts. DTF shows high outgoing connections from one of the contacts in the right anterior hippocampus (RHA1). At the end of the seizure the connectivity pattern resembles a bit more the resting state network, albeit a more interconnected version, with mostly intra-regional connections. The connectivity matrices from the final epoch at the end of the recording also show a resemblance with those obtained from the pre-stimulation epoch, especially for GCI and CGCI.

**Figure 8:**
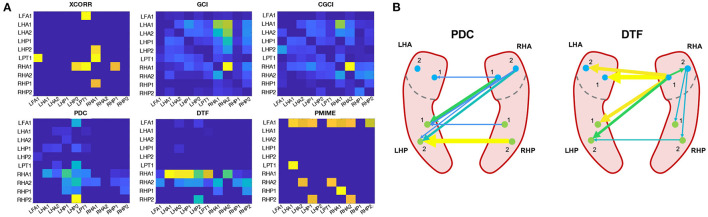
Connectivity results from the sEEG epoch at the beginning of the seizure (*t* = 166 − 168 s). **(A)** Connectivity matrices for each connectivity measure. **(B)** Simplified schematic representation of the intra- and inter-hippocampal networks based on the PDC and DTF results.

We will look a bit more in detail at the epoch at the beginning of the seizure, because this is the one expected to best represent the actual ictal network. The connectivity matrices and a schematic reconstruction of the hippocampal network during seizure are presented in Figure 8. The GCI and CGCI results are very similar, with a main direction of information flow from the left to the right hippocampus. The two most prominent interactions in the CGCI network are LHA1 → RHA1 and RHA1 → RHA2. PDC and DTF both return networks with a directionality from the right to the left hippocampus. DTF puts an emphasis on the outgoing connections from the RHA1 contact. Figure 8B contains a simplified schematic representation of the intra- and inter-hippocampal networks as returned by PDC and DTF.

The connectivity matrices returned by cross-correlation and PMIME show no clear structure, and both contain outgoing connections from the left frontal contact (LFA1) toward other contacts in the left hemisphere.

**Figure 9:**
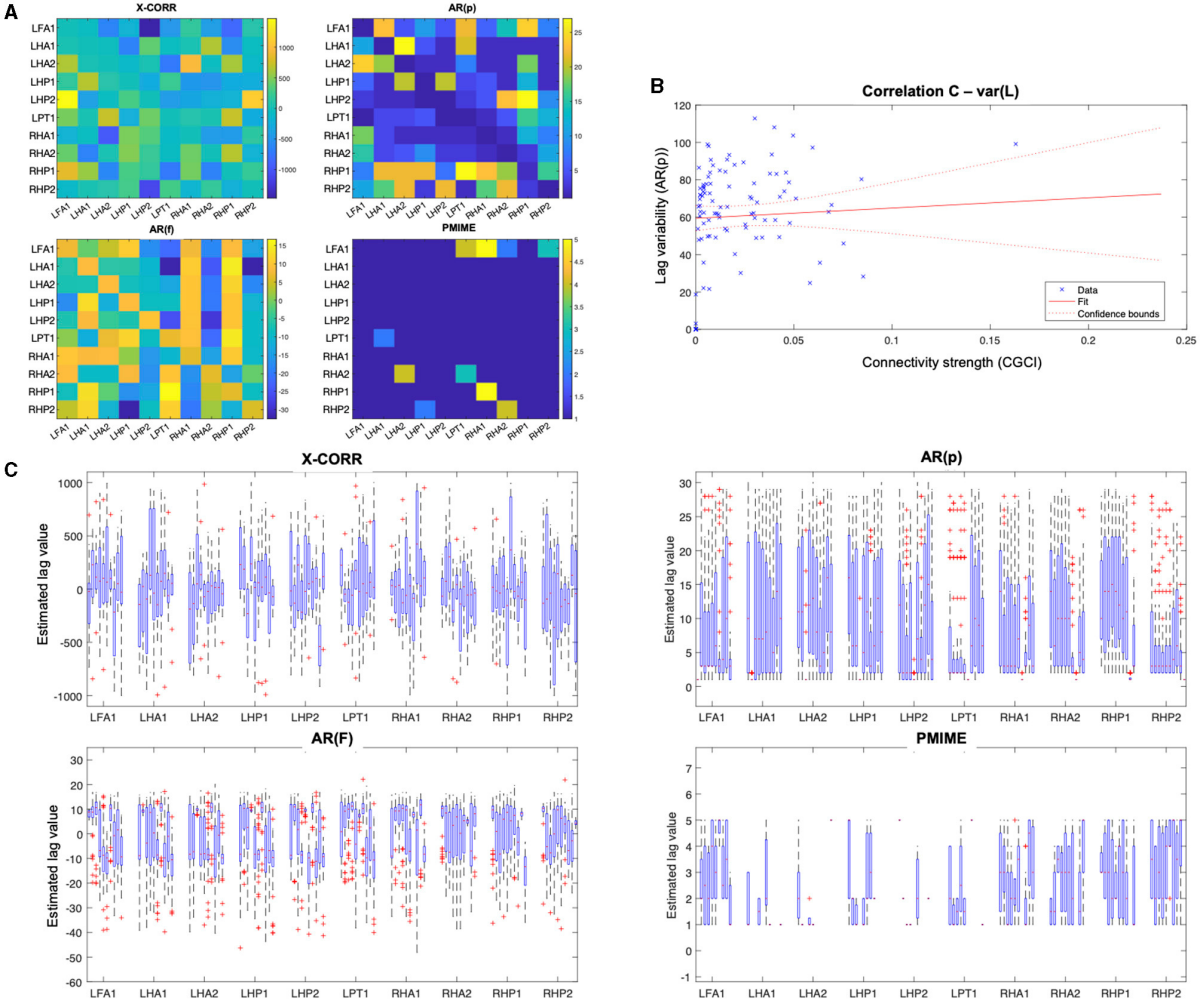
Lag estimation results from the sEEG seizure data. **(A)** Lag estimation at the beginning of the seizure (*t* = 166 − 168 s). **(B)** Correlation plot of the lag variability during the seizure in function of the connectivity strength, as estimated by the AR(p) and CGCI measures, respectively. Each blue cross represents a data point, the red line shows a linear fitted model with the 95%-confidence bounds as dotted lines. **(C)** Box plot of the lag values for each channel pair, estimated during the entire seizure.

The lag estimation results are displayed in Figure 9. For the lag matrices, each element (i,j) represents the estimated lag value of the connection from contact i to j. Because the lag values right after the seizure onset are hard to interpret and show no clear pattern, we also took a closer look at the variability of the lag estimations. Figure 9C shows the box plots of the lag values estimated from epochs taken throughout the entire seizure. The box plots are grouped into clusters containing the values for outgoing connections from the contact that is indicated on the x-axis. Within each cluster, the same order of contacts is used i.e., the first box shows the lags from LFA1 → LFA1, the second box from LFA1 → LHA1, and so on. These plots clearly show the large variance and apparent absence of a clear pattern in the lag values. The correlation plot in Figure 9C shows the interaction between the CGCI connectivity strength and the AR(p) lag variability.

## 7. Discussion

### 7.1. Causality Measures

The large overlap between the MCC distributions for the causality measures indicates that there's no one measure that clearly performs better in all contexts. It is therefore informative to check how the measures relate to each other at the level of the simulation models. Here we see a surprisingly bad performance on the simplest simulation model (“Random”), while the best results are seen for “Henon.” This difference could be explained by the stronger internal structure of the signals in the latter model, whereas in the “Random” model the signals essentially consist of pure noise. For the linear AR models and the AR models with frequency-specific interactions PDC and DTF give the best performance, with similar results obtained using GCI or CGCI. However, if we take these measures outside of their comfort zone and analyse chaotic models or introduce non-linear interactions, they are significantly outperformed by PMIME. These results indicate the importance of choosing a causality measure based on the expected characteristics of the system that's being analysed.

With the average connectograms we can go into even more detail and investigate if and where in the different systems there is a tendency to consistently make the same mistake. With its long cascade of information transfer, the “CascadeAR” model lends itself perfectly to show the influence of indirect links on the different measures. While the network of the bivariate GCI is severely impacted by the presence of these indirect connections, its multivariate extension CGCI is able to better tell the difference between direct and spurious indirect connections. At the same time it can also be seen that DTF finds these indirect connections as well, mostly toward variable *X*_3_ which is a sink in the network. This leads to a lower MCC value for DTF, even though these are not really false positives as DTF is meant to find these indirect connections (see section 3.5).

Looking at the reconstructed networks for the “PinkARlin” models, those from PDC, DTF and PMIME clearly correspond most to the ground truth. If we then introduce non-linear interactions in “PinkARnonlin” we see that only PMIME retains its good performance. This was expected since its non-parametric nature makes it the only metric that's able to detect non-linear interactions. However, what is unexpected is that the other measures return completely disrupted networks. Our hypothesis was that these AR-based metrics would only miss non-linear connections but maintain their performance in the rest of the network. These results now indicate that the effect of non-linearities can be propagated throughout the entire network and lead to a significant increase in false positives. It is therefore important to always interpret results from these AR-based metrics with caution, especially when dense networks are returned.

**Figure 10:**
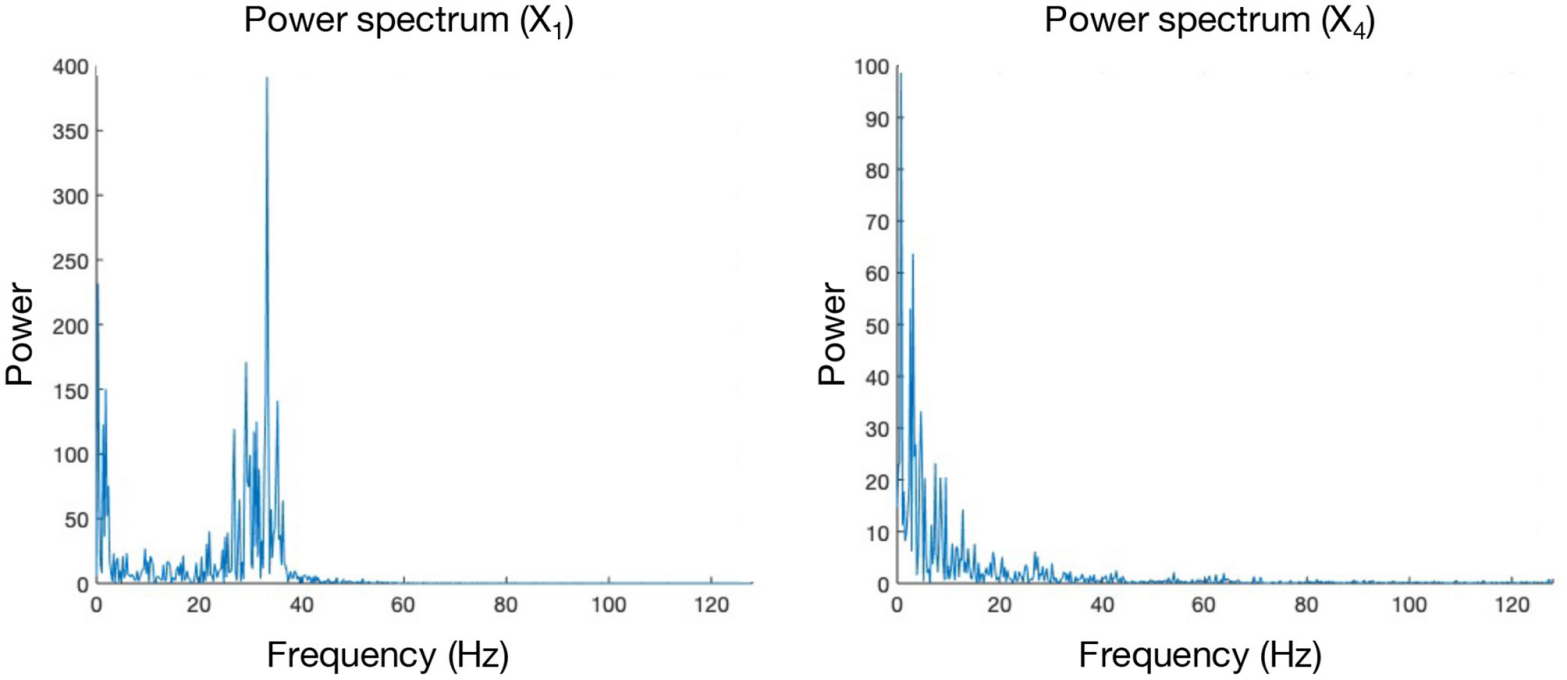
Power spectra of signals *X*_1_ and *X*_4_, obtained from a realisation of the “FreqARlin” simulation model.

Even though PMIME seems to perform well on all previously mentioned models, it doesn't do well in the presence of frequency-specific connections. The mechanism behind why PMIME performs this poorly in this context is not completely clear and will require some further investigation in the future. However, this issue is presumed to be mediated by the reconstruction of separate functional networks for each frequency band of interest. In the simulation models that contain frequency-specific connections, the other causality measures (GCI, CGCI, DTF, and PDC) give the best performance. Here PDC and DTF seem the more logical choice as a causality measure, since the frequency-dependency of the connections is already embedded in its computations and can also be extracted from the results. Notably, none of the measures is able to detect the low-frequency connection *X*_1_ → *X*_4_. This can be explained by looking at the spectral characteristics of both signals. Whereas the power spectrum of *X*_4_ follows a simple 1/*f* distribution, *X*_1_ has an additional peak around 35 Hz. If the connection *X*_1_ → *X*_4_ were to occur in the γ frequency band (i.e., [25–100 Hz]), this peak in the spectrum of *X*_1_ would lead to a large impact on *X*_4_ as the gamma power of the latter is close to zero. The larger the impact, the easier it will be to correctly detect this interaction. This effect will however be less for low-frequency connections, and therefore the coupling is more difficult to detect Figure 10.

### 7.2. Lag Estimation

The lag estimation models were evaluated on a set of simulation models with variable lag and designed to cover a range of characteristics: periodic and stochastic processes generating bivariate and multivariate systems with linear, non-linear, and frequency-specific couplings. As the AR(f) method seems to randomly select lags around zero in all cases, this model is clearly unable to detect the lag between communicating variables. Cross-correlation can be used to reliably detect the lag in bivariate systems except with frequency-specific connections, albeit sometimes with the wrong directionality. The AR(p) method performs well in all linear cases, bivariate as well as multivariate, and is the only one able to find the correct lag in the frequency-specific model. These results are in line with what we saw in the connectivity results, where the AR metrics could detect the correct network in the FreqARlin while PMIME performed poorly in this context. As expected, only the PMIME based lag estimation performs well also for non-linear couplings.

Due to its easy and fast computation AR(p) presents itself as an ideal candidate to identify the lag between coupled variables, as long as the interaction is linear. If the coupling is non-linear, the correct lag can be identified with PMIME. The latter can also be used in all other contexts as long as the interaction is not frequency-specific, however computation is more complex and time-consuming.

### 7.3. Application on sEEG Seizure Data

When we look at the results from all connectivity measures on all sEEG epochs (see Figure 7) it's clear that the AR-based measures all return similar networks. This similarity can be expected and is largest between GCI and CGCI. The difference with their frequency domain counterparts (i.e., PDC and DTF) is larger, their networks show the same overall patterns but are more sparse. They appear to return more sharply defined networks, which allows for a focussed analysis of the important connections at play.

During stimulation we see a sharp transition in the network structure with strong inter-hippocampal connections from the right to the left hippocampus (except for GCI which shows more a left-to-right directionality, but this is probably erroneous and caused by the periodicity of the signals). This hyper-synchrony caused by the stimulation is a very probable driving factor in the development of the seizure activity, and the directionality from the right to the left hippocampus is in agreement with what we expected based on the location of the stimulation contacts (RA1/2).

At the beginning of the seizure the networks are sparser and more unstructured. This might seem counter-intuitive since epileptic seizures are associated with hyper-synchrony, but desynchronisation is often observed right before and after the onset of ictal activity (Mormann et al., 2003; Schiff et al., 2005; Schindler et al., 2010). Other analyses have reported increased connectivity at seizure onset (Schindler et al., 2007; Kramer et al., 2010). Differences between these results may arise from a multitude of reasons including seizure type, coupling measure, electrode locations etc. The exact mechanisms of brain functional connectivity at the seizure onset are not completely understood and remain an active area of research. Further discussion of the hippocampal seizure network will be given in the next paragraph.

At the end of the seizure the networks are partly restored and resemble a more densely connected version of the resting state network. High levels of synchronisation are often observed in late stages of the seizure and may play a role in facilitating termination of the seizure (Schindler et al., 2007). For an extensive overview and discussion on the dynamics of functional connectivity during seizure progression, we recommend the review from Kramer and Cash (2012).

At the end of the recording, when there is no longer seizure activity and/or stimulation artifacts present in the signal, the connectivity networks again resemble those obtained from the pre-stimulation epoch. The resemblance is most strong for the GCI and CGCI networks, which might therefore be the most suitable candidates for resting state connectivity analysis in EEG. The pre-stimulation and post seizure connectivity matrices are expected to show some differences, because resting state networks are variable in time.

The choice of the epoch length always has an impact on the results. Too short epochs don't contain sufficient data points to fit the models while too long epochs might contain non-stationary behaviour, which EEG signals are notorious for. We investigated the effect of the epoch length on our connectivity results by comparing the connectivity matrices for epoch lengths of 0.5, 1, 2, 3, and 5 s. The results of this analysis can be seen in Supplementary Figures 2–5. For short epochs the metrics are no longer able to detect all relationships and the returned networks are sparser, especially for PDC and DTF. GCI, a bivariate model, shows more stable results when the epochs become shorter due to the lower number of parameters that need to be fitted. With longer epoch lengths we see the strongest difference in the networks from the pre-stimulation epoch, which may be assumed to contain the most non-stationary behaviour. The epoch during stimulation, dominated by the constant stimulatory component, is mostly stationary. These networks are less impacted by an increased epoch length. The results for epoch lengths 2 and 3 s are very similar and the optimal epoch length is therefore expected to lie within this range, justifying our choice of 2 s.

Because the epoch right after the seizure onset best represents the seizure network, these networks are analysed a bit more in-depth (see Figure 8). The networks returned by GCI and CGCI are very similar, with CGCI returning a slightly sparser version of the GCI network. Both measures indicate an information flow from the left to the right anterior hippocampus, mainly through the link LHA1 → RHA1. This directionality is contradictory to our expectations, as the seizure activity clearly starts in the right hippocampus (see Figure 2). Apart from the unexpected directionality, the role of the RHA1 contact is in line with our findings on the source analysis with the spectral measures (PDC and DTF). These measures clearly detect the right-to-left-directionality of information flow and return very plausible reconstructions of the ictal network. From the PDC network it is already apparent that the largest information spread originates in the right anterior hippocampus (RHA1 and RHA2), but it is hard to see which of the two contacts is driving the ictal network. DTF on the other hand clearly identifies RHA1 as the main source. This concurs with our observations of the local field potentials during the seizure onset. The random nature of the cross-correlation connectivity matrix further confirms its poor performance as a connectivity measure, as we also saw in the results from the simulation models. PMIME also returns a network that's very distinct from those of the AR-based methods. This could indicate the presence of a network with non-linear interactions, but the strong outgoing connections from the LAF1 contact are hard to explain and suggest an erroneous network. This might be caused by the frequency-specific nature of the connections, which showed to cause wrong outputs from PMIME in the simulation models. This is also in line with the hypothesis of frequency-specific connections in brain communication.

At first sight the lag estimation shows no clear pattern that relates to the connectivity network found during seizure onset. The large variance of the lag estimations during the seizure, seen in the box plots, suggests there's no stable relationship between the connectivity and lag estimation results. This is further endorsed by the lack of correlation between the connectivity strength and lag variability. These results are inconsistent with how we approach connectivity in the brain and raise some important questions. Based on the neuron doctrine, a line of thinking has emerged where connections between brain regions have been regarded as a causal influence of region A on region B with a certain delay related to the propagation of action potentials across axons and synapses. However, when network structures become more complex with back-connections, self-connections, loops etc. this concept of causality becomes less clear and other models (e.g., chaotic systems) might be more fitting. The apparent independence of the lag and connectivity strength needs to be further investigated to check whether this is related to unreliability of the lag estimation methods or indeed we have to rethink our conceptual approach on brain connectivity.

### Limitations and Future Perspectives

In this paper we compare a set of popular causality measures in simulated systems in an attempt to characterise their performance in different environments. However, this is not an exhaustive comparison and is not aimed at providing a single answer on how to perform causality analysis on a given dataset. Many other causality measures and adaptations thereof exist that can be combined with different statistical tests.

Several simulation models were used in an attempt to reproduce the signal characteristics that we might encounter in true observations. Some models are focused on recreating the spectral behaviour (e.g., the 1/*f* spectral distribution of all AR signals), while others are aimed at simulating different possible coupling characteristics (linear, non-linear, and/or frequency-specific). Simulation models such as “Sweep” and “FreqARlin” were designed to produce specific clinically relevant signal attributes based on theoretical principles of brain networks (i.e., the spreading of epileptic seizures and frequency-specific communication between brain regions, respectively). The resulting signals may sometimes diverge from what we see in reality, but all models were designed and chosen carefully to provide as much insight as possible in how the causality measures are affected by and handle different types of data.

The effect of changes in SNR, signal length, number of channels and sparsity of the network were not taken into account here, and sensitivity tests to these parameters might very well lead to other relevant insights. Also, the simulation models used here were designed to resemble EEG signals, but other conclusions may hold for signals with very different characteristics (e.g., low temporal resolution signals such as in functional magnetic resonance imaging).

We applied the same methods to sEEG signals to get an impression of how the conclusions of the simulation study can be translated to real-world applications. However, we want to stress that this is only one example and connectivity results in other analyses should always be interpreted with caution.

## 8. Conclusion

This work consists of three main components. First, we provided an extensive evaluation of some of the most known causality measures that are being used in the context of functional connectivity analysis. Based on a set of simulation models with distinct characteristics (chaotic vs. stochastic processes, linear vs. non-linear, and general vs. frequency-specific couplings) we were able to show the context-dependent ability of these measures to correctly reconstruct the underlying network. For multivariate systems of a stochastic nature with purely linear interactions, PDC and DTF performed best. Information-theory based measures such as PMIME are preferred for chaotic systems and/or if non-linear interactions are present. Notably, PMIME performed poorly when the interactions are frequency-specific. Another interesting result is that the effects non-linear couplings seem to, in stochastic systems, propagate through-out the entire network and completely disrupt the results from linear measures such as PDC and DTF. This issue warrants the use of both linear and non-linear causality measures when examining data of an unknown nature (e.g., for the analysis of physiological data such as EEG). If the reconstructed networks differ strongly and the network returned by the linear measure is very dense, this suggests the presence of non-linearities and advocates caution when interpreting the results from the linear measure. Table 1 contains an overview of the main conclusions from the simulation models on when to apply which measure. These conclusions may serve as an indication of which causality measure is best fitted for the expected signal characteristics but should under no circumstance be interpreted as a plug-and-play guide to choose one metric. Because of the bad results of PMIME on the models with frequency-specific interactions, further investigation is needed to find a suitable candidate for connectivity analysis in systems with non-linear, frequency-specific interactions.

**Table 1:** Overview of the different signal characteristics covered in this paper, in terms of generative processes and interaction types, and the causality measures that were found to perform best under these circumstances in the simulation models.

		**Linear**	**Non-linear**
Stochastic	General	CGCI or PDC for network reconstruction, DTF to find the network sources. CGCI can be used to get a more dense representation of the network, which might be helpful to compute graph measures.	PMIME
	Frequency-specific	PDC for network reconstruction, DTF to find the network sources.	???
Periodic		PDC for network reconstruction, DTF to find the network sources. Beware for directionality issues.	PMIME
Chaotic		PMIME	PMIME

*This is only a general indication and should not be interpreted as a plug-and-play roadmap for selecting a causality measure*.

In the second part of this study we proposed and evaluated four methods to estimate the lag between coupled variables. The PMIME-based method was shown to be an excellent lag estimator and correctly identified the true lag for all realisation of most models, even to some extent for frequency-specific connections. Using the magnitude of the (time-domain) AR coefficients also detected the correct lag, but only for models with purely linear interactions. Therefore, the PMIME-based lag estimation is preferred over the AR(p) method, unless the system is known to only contain linear interactions. In this case the AR(p) method might be preferred as its easier and faster to compute. Also due to its computation-expensive nature PMIME poses a limit on the number of variables in the system and the depth of the embedding dimension that can be used (i.e., how many samples we can look back in time).

In the end, we applied the same connectivity measures and lag estimation methods on a sEEG recording of a stimulation-induced epileptic seizure. Overall, the functional connectivity results show a good correspondence with the results from the simulation models. The AR-based spectral methods returned a plausible network that agrees with the observations of the measured signals and known concepts of desynchronisation and synchronisation during seizure progression. The lag estimation results show an unexpected independence between the estimated lag and connectivity strength. Further investigation is required to check if we need to fine-tune the lag metrics, or rethink our concepts of causality in regard to the complex dynamics of brain networks.

## Data Availability Statement

The datasets presented in this article are not readily available because of privacy issues. The code for the simulation models can however be provided upon request. Requests to access the datasets should be directed to jolan.heyse@ugent.be.

## Ethics Statement

The studies involving human participants were reviewed and approved by Geneva University Hospitals (HUG), Geneva, Switzerland. The patients/participants provided their written informed consent to participate in this study.

## Author Contributions

JH and PM planned and designed the study. SV and LS acquired the clinical data. JH analysed and interpreted the data and drafted the manuscript. All the authors revised and approved the manuscript.

## Funding

This work was supported by the Research Foundation Flanders - FWO under Grant 1S87220N (SB PhD fellowship, JH), by the Swiss National Science Foundation grants no. CRSII5 180365 (PM), CRSII5 170873 (PvM and SV), and SNF 192749 (SV). LS was funded by a grant from the Faculty of Medicine, University of Geneva (“médecin interne scientifique”).

## Conflict of Interest

PM and SV are shareholders of Epilog NV. The remaining authors declare that the research was conducted in the absence of any commercial or financial relationships that could be construed as a potential conflict of interest.

## Publisher's Note

All claims expressed in this article are solely those of the authors and do not necessarily represent those of their affiliated organizations, or those of the publisher, the editors and the reviewers. Any product that may be evaluated in this article, or claim that may be made by its manufacturer, is not guaranteed or endorsed by the publisher.

## Footnotes

^1^sEEG is an invasive procedure where electrodes are implanted in targeted brain areas to identify where epileptic seizures originate.

^2^A violin plot is a hybrid visualisation technique that contains the same statistics of a box plot (i.e., median and interquartile ranges), with additional information on the probability density function represented by the width of the box. The probability density function is estimated using a Gaussian kernel density estimator.
