# Supplementary figures and images for: Evaluation of Directed Causality Measures and Lag Estimations in Multivariate Time-Series
Source: Front Syst Neurosci. 2021 Oct 22;15:620338. doi: 10.3389/fnsys.2021.620338 (PMC8569855; doi:10.3389/fnsys.2021.620338)

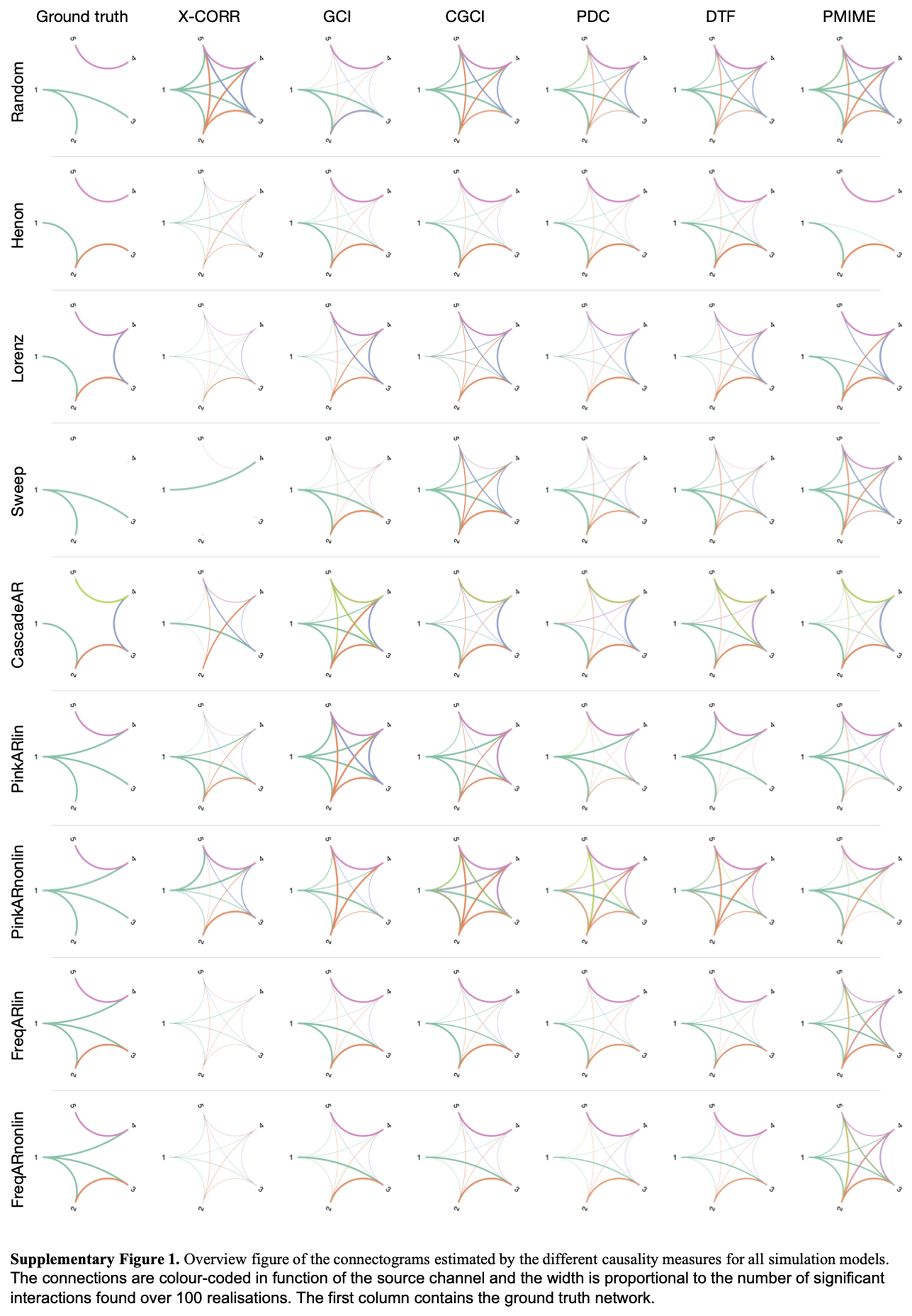

Supplement: Supplementary file 1 [file Image_1.tiff]

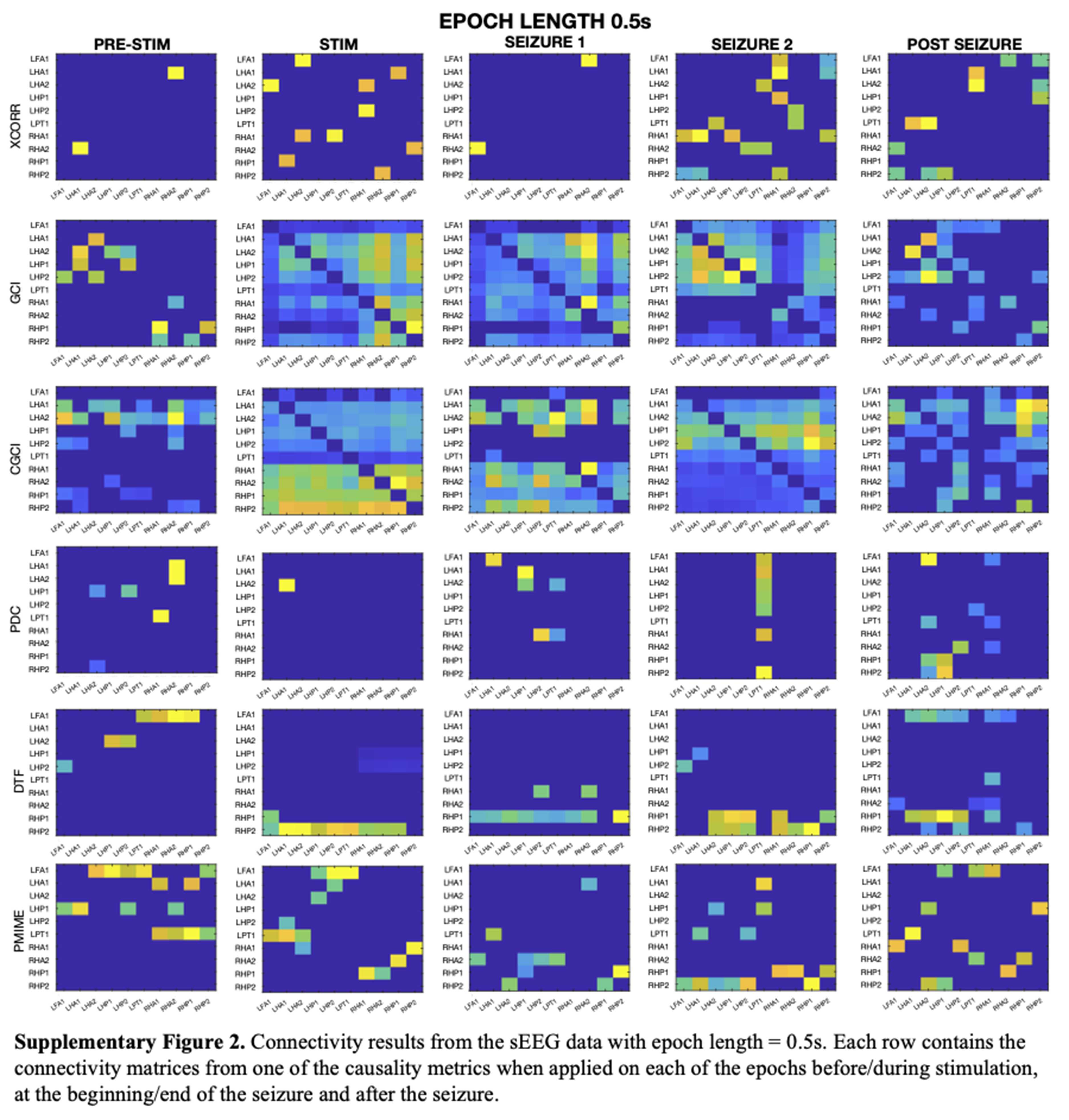

Supplement: Supplementary file 2 [file Image_2.tiff]

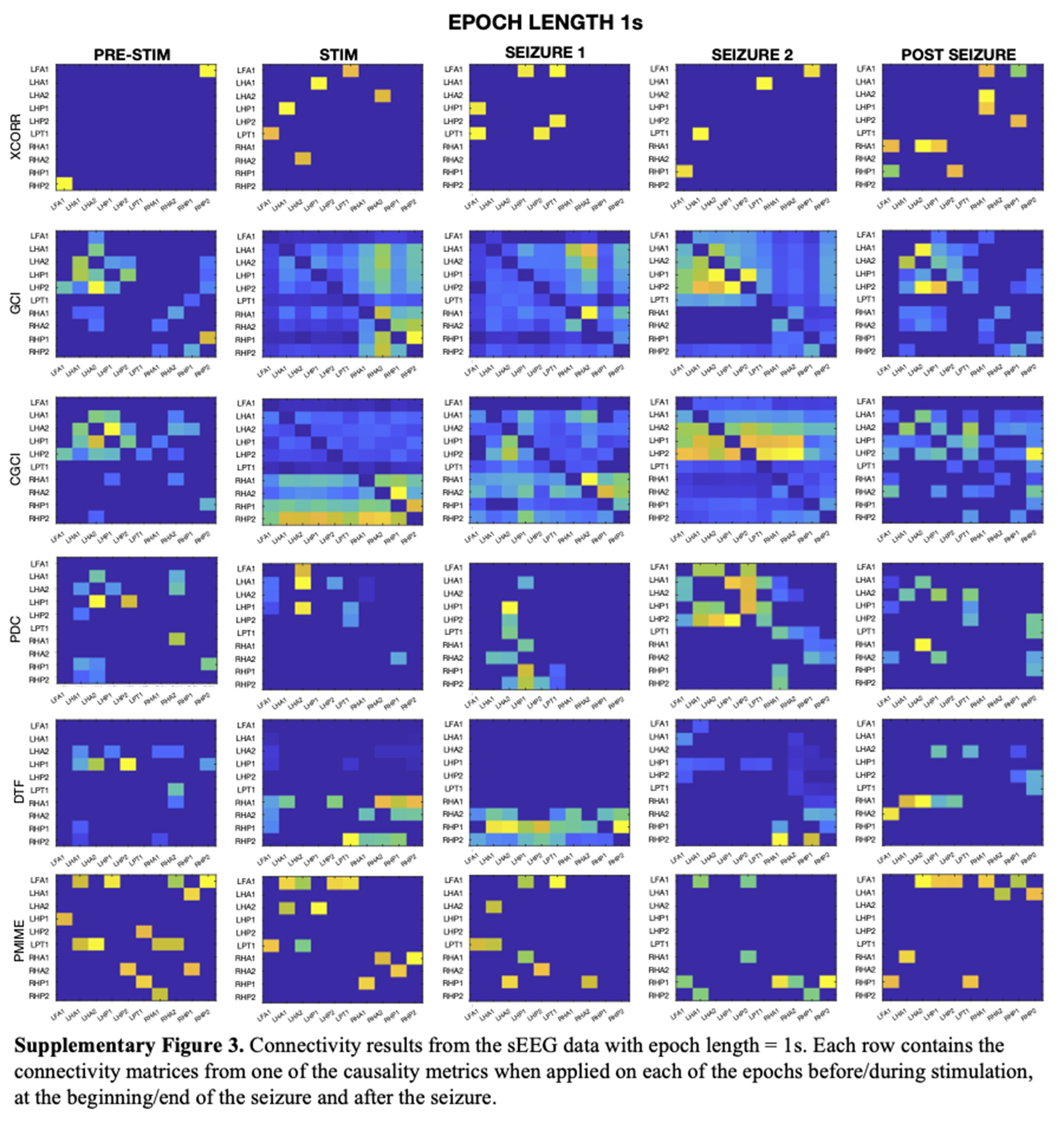

Supplement: Supplementary file 3 [file Image_3.tiff]

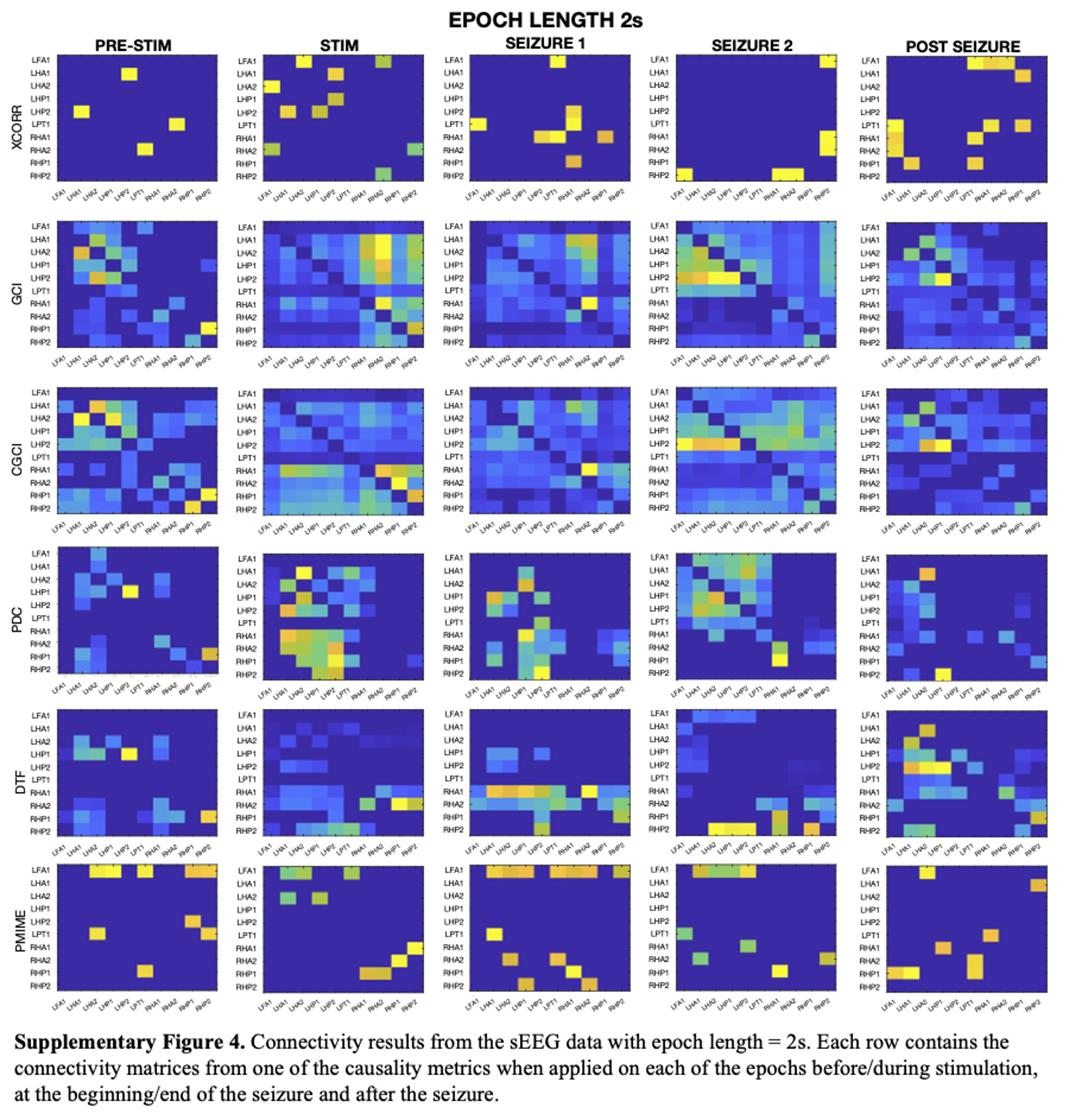

Supplement: Supplementary file 4 [file Image_4.tiff]

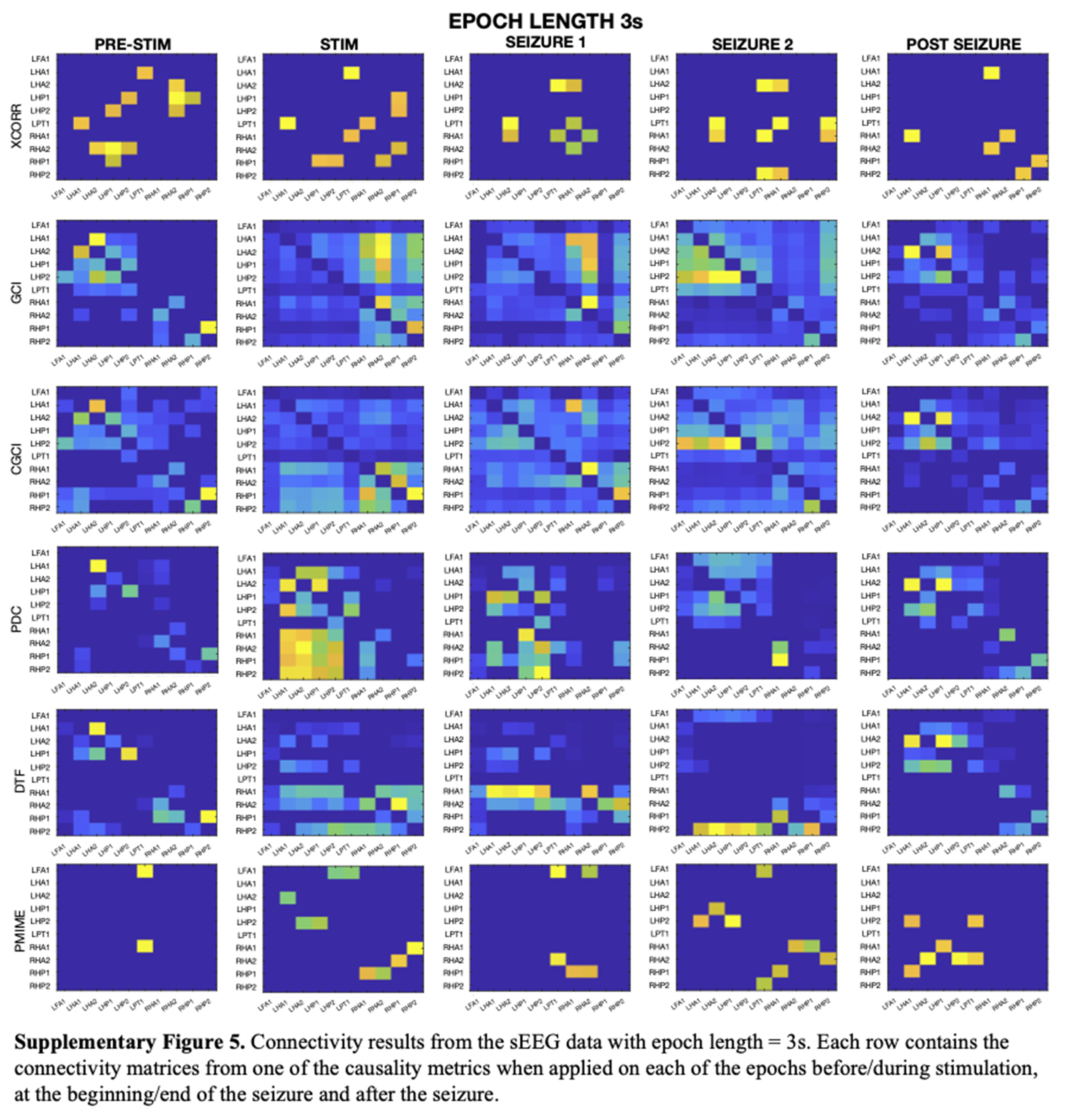

Supplement: Supplementary file 5 [file Image_5.tiff]

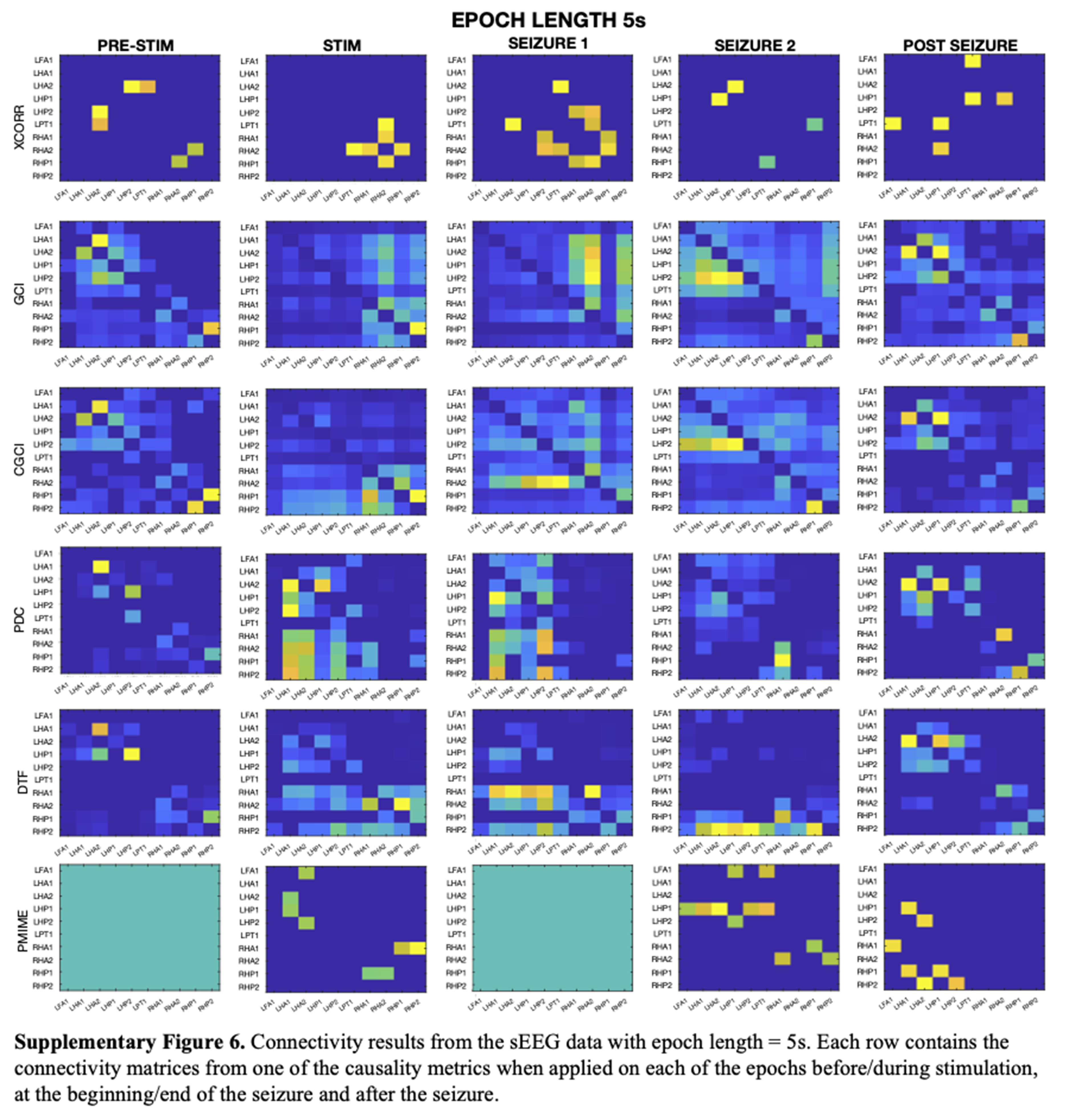

Supplement: Supplementary file 6 [file Image_6.tiff]
