# Supplementary material for: Evaluation of Directed Causality Measures and Lag Estimations in Multivariate Time-Series
Source: Front Syst Neurosci. 2021 Oct 22;15:620338. doi: 10.3389/fnsys.2021.620338 (PMC8569855; doi:10.3389/fnsys.2021.620338)
Supplement: Supplementary file 7 [file Table_1.DOCX]

| **SENS/PREC** | Random | Henon | Lorenz | Sweep | CascadeAR | PinkARlin | PinkARnonlin | FreqARlin | FreqARnonlin |
| --- | --- | --- | --- | --- | --- | --- | --- | --- | --- |
| X-CORR | 99/15 | 11/13 | 20/40 | 0/0 | 11/11 | 51/35 | 71/31 | 9/33 | 6/25 |
| GCI | 100/57 | 100/67 | 71/48 | 100/30 | 100/52 | 100/28 | 76/38 | 81/59 | 61/61 |
| CGCI | 100/21 | 100/72 | 70/42 | 100/18 | 100/77 | 100/50 | 78/26 | 75/60 | 59/63 |
| PDC | 100/35 | 100/75 | 60/61 | 100/43 | 99/83 | 100/83 | 62/34 | 62/67 | 46/70 |
| DTF | 100/28 | 100/62 | 60/51 | 100/38 | 76/64 | 99/78 | 75/35 | 69/73 | 50/69 |
| PMIME | 100/18 | 100/97 | 100/70 | 100/22 | 100/83 | 82/63 | 86/71 | 64/36 | 64/36 |

**Supplementary Table 1.** Sensitivity and precision, expressed in percentages, for each possible combination of the causality measures and simulation models.
