# Supplementary material for: Evaluation of Directed Causality Measures and Lag Estimations in Multivariate Time-Series
Source: Front Syst Neurosci. 2021 Oct 22;15:620338. doi: 10.3389/fnsys.2021.620338 (PMC8569855; doi:10.3389/fnsys.2021.620338)
Supplement: Supplementary file 8 [file Table_2.DOCX]

| **MCC** | Random | Henon | Lorenz | Sweep | CascadeAR | PinkARlin | PinkARnonlin | FreqARlin | FreqARnonlin |
| --- | --- | --- | --- | --- | --- | --- | --- | --- | --- |
| X-CORR | 0,18 | 0,01 | 0,20 | -0,09 | -0,18 | 0,24 | 0,26 | 0,07 | 0,03 |
| GCI | 0,72 | 0,79 | 0,49 | 0,49 | 0,60 | 0,31 | 0,37 | 0,60 | 0,51 |
| CGCI | 0,32 | 0,82 | 0,43 | 0,33 | 0,83 | 0,62 | 0,19 | 0,58 | 0,51 |
| PDC | 0,51 | 0,85 | 0,53 | 0,62 | 0,88 | 0,89 | 0,27 | 0,56 | 0,49 |
| DTF | 0,43 | 0,75 | 0,46 | 0,57 | 0,59 | 0,84 | 0,33 | 0,64 | 0,51 |
| PMIME | 0,27 | 0,99 | 0,80 | 0,39 | 0,88 | 0,64 | 0,72 | 0,29 | 0,29 |

**Supplementary Table 2.** Mathew's correlation coefficient values for each possible combination of the causality measures and simulation models.
