# Supplementary material for: Evaluation of Directed Causality Measures and Lag Estimations in Multivariate Time-Series
Source: Front Syst Neurosci. 2021 Oct 22;15:620338. doi: 10.3389/fnsys.2021.620338 (PMC8569855; doi:10.3389/fnsys.2021.620338)
Supplement: Supplementary file 9 [file Table_3.DOCX]

|  |  | **GCI** | | | **CGCI** | | | **PDC** | | | **DTF** | | | **PMIME** |
| --- | --- | --- | --- | --- | --- | --- | --- | --- | --- | --- | --- | --- | --- | --- |
|  |  | p = 2 | p = 5 | p = 10 | p = 2 | p = 5 | p = 10 | p = 2 | p = 5 | p = 10 | p = 2 | p = 5 | p = 10 | Lmax = 5 |
| **Henon** | n = 125 | 0,52 | 0,42 | 0,47 | 0,24 | 0,16 | 0,13 | 0,19 | 0,14 | 0,14 | 0,16 | 0,13 | 0,20 | 0,62 |
|  | n = 250 | 0,77 | 0,80 | 0,81 | 0,57 | 0,60 | 0,55 | 0,49 | 0,48 | 0,46 | 0,46 | 0,45 | 0,43 | 0,94 |
|  | n = 500 | 0,85 | 0,84 | 0,85 | 0,83 | 0,85 | 0,83 | 0,85 | 0,85 | 0,89 | 0,84 | 0,78 | 0,82 | 0,99 |
|  | n = 1000 | 0,81 | 0,80 | 0,78 | 0,83 | 0,82 | 0,81 | 0,79 | 0,86 | 0,84 | 0,77 | 0,76 | 0,77 | 1,00 |
| **Lorenz** | n = 125 | 0,31 | 0,31 | 0,31 | 0,20 | 0,18 | 0,19 | 0,28 | 0,24 | 0,26 | 0,26 | 0,24 | 0,22 | 0,44 |
|  | n = 250 | 0,30 | 0,34 | 0,32 | 0,22 | 0,23 | 0,21 | 0,33 | 0,30 | 0,31 | 0,30 | 0,23 | 0,31 | 0,59 |
|  | n = 500 | 0,33 | 0,39 | 0,39 | 0,30 | 0,35 | 0,30 | 0,34 | 0,40 | 0,35 | 0,35 | 0,40 | 0,35 | 0,75 |
|  | n = 1000 | 0,45 | 0,47 | 0,48 | 0,45 | 0,46 | 0,45 | 0,46 | 0,51 | 0,51 | 0,49 | 0,48 | 0,46 | 0,80 |
| **Sweep** | n = 125 | 0,04 | 0,00 | 0,00 | 0,02 | 0,03 | 0,01 | 0,03 | -0,01 | 0,01 | -0,02 | 0,01 | 0,00 | 0,10 |
|  | n = 250 | 0,03 | 0,02 | 0,02 | 0,00 | 0,00 | 0,00 | 0,00 | 0,04 | 0,00 | 0,04 | 0,02 | 0,00 | 0,14 |
|  | n = 500 | 0,67 | 0,60 | 0,59 | 0,51 | 0,53 | 0,50 | 0,48 | 0,45 | 0,45 | 0,86 | 0,84 | 0,84 | 0,27 |
|  | n = 1000 | 0,50 | 0,49 | 0,49 | 0,38 | 0,32 | 0,33 | 0,50 | 0,61 | 0,60 | 0,65 | 0,58 | 0,61 | 0,38 |
| **PinkAR- lin** | n = 125 | 0,35 | 0,41 | 0,44 | 0,70 | 0,71 | 0,67 | 0,66 | 0,61 | 0,61 | 0,65 | 0,63 | 0,64 | 0,37 |
|  | n = 250 | 0,31 | 0,40 | 0,39 | 0,64 | 0,63 | 0,65 | 0,78 | 0,72 | 0,75 | 0,66 | 0,68 | 0,67 | 0,44 |
|  | n = 500 | 0,28 | 0,33 | 0,34 | 0,56 | 0,68 | 0,66 | 0,81 | 0,84 | 0,86 | 0,70 | 0,75 | 0,75 | 0,55 |
|  | n = 1000 | 0,27 | 0,29 | 0,30 | 0,46 | 0,60 | 0,61 | 0,74 | 0,89 | 0,87 | 0,69 | 0,84 | 0,84 | 0,65 |
| **PinkAR-nonlin** | n = 125 | 0,36 | 0,39 | 0,36 | 0,28 | 0,20 | 0,19 | 0,15 | 0,14 | 0,16 | 0,11 | 0,16 | 0,08 | 0,42 |
|  | n = 250 | 0,36 | 0,40 | 0,38 | 0,23 | 0,20 | 0,20 | 0,15 | 0,19 | 0,21 | 0,13 | 0,20 | 0,21 | 0,58 |
|  | n = 500 | 0,33 | 0,38 | 0,40 | 0,26 | 0,19 | 0,22 | 0,15 | 0,27 | 0,27 | 0,12 | 0,27 | 0,25 | 0,66 |
|  | n = 1000 | 0,33 | 0,36 | 0,38 | 0,26 | 0,18 | 0,22 | 0,16 | 0,24 | 0,25 | 0,10 | 0,34 | 0,31 | 0,75 |
| **CascadeAR** | n = 125 | 0,79 | 0,81 | 0,79 | 0,84 | 0,74 | 0,72 | 0,79 | 0,57 | 0,51 | 0,50 | 0,42 | 0,45 | 0,40 |
|  | n = 250 | 0,76 | 0,70 | 0,71 | 0,77 | 0,77 | 0,78 | 0,92 | 0,64 | 0,61 | 0,57 | 0,46 | 0,45 | 0,51 |
|  | n = 500 | 0,68 | 0,63 | 0,65 | 0,73 | 0,81 | 0,80 | 0,96 | 0,81 | 0,82 | 0,65 | 0,52 | 0,49 | 0,66 |
|  | n = 1000 | 0,61 | 0,62 | 0,60 | 0,67 | 0,83 | 0,83 | 0,94 | 0,90 | 0,92 | 0,71 | 0,59 | 0,58 | 0,90 |
| **FreqAR- lin** | n = 125 | 0,18 | 0,34 | 0,27 | 0,25 | 0,21 | 0,22 | 0,05 | 0,07 | 0,09 | 0,09 | 0,15 | 0,11 | 0,20 |
|  | n = 250 | 0,31 | 0,45 | 0,46 | 0,42 | 0,49 | 0,49 | 0,23 | 0,38 | 0,29 | 0,21 | 0,33 | 0,36 | 0,24 |
|  | n = 500 | 0,54 | 0,58 | 0,57 | 0,55 | 0,56 | 0,56 | 0,34 | 0,47 | 0,49 | 0,42 | 0,53 | 0,56 | 0,27 |
|  | n = 1000 | 0,65 | 0,61 | 0,60 | 0,61 | 0,57 | 0,57 | 0,49 | 0,58 | 0,56 | 0,57 | 0,60 | 0,64 | 0,28 |
| **FreqAR-nonlin** | n = 125 | 0,19 | 0,17 | 0,17 | 0,23 | 0,15 | 0,17 | 0,10 | 0,05 | 0,07 | 0,11 | 0,12 | 0,10 | 0,23 |
|  | n = 250 | 0,22 | 0,29 | 0,24 | 0,33 | 0,34 | 0,41 | 0,18 | 0,27 | 0,27 | 0,19 | 0,25 | 0,23 | 0,23 |
|  | n = 500 | 0,39 | 0,44 | 0,45 | 0,45 | 0,49 | 0,48 | 0,34 | 0,42 | 0,40 | 0,33 | 0,39 | 0,41 | 0,25 |
|  | n = 1000 | 0,47 | 0,53 | 0,52 | 0,49 | 0,55 | 0,54 | 0,41 | 0,50 | 0,54 | 0,41 | 0,50 | 0,49 | 0,30 |
| **Random** | n = 125 | 0,29 | 0,71 | 0,70 | 0,02 | 0,00 | 0,02 | 0,06 | 0,05 | -0,01 | 0,11 | 0,10 | 0,09 | 0,24 |
|  | n = 250 | 0,29 | 0,71 | 0,73 | 0,19 | 0,19 | 0,21 | 0,20 | 0,28 | 0,28 | 0,40 | 0,43 | 0,42 | 0,25 |
|  | n = 500 | 0,28 | 0,72 | 0,72 | 0,20 | 0,30 | 0,31 | 0,23 | 0,51 | 0,50 | 0,43 | 0,42 | 0,43 | 0,26 |
|  | n = 1000 | 0,28 | 0,74 | 0,73 | 0,18 | 0,32 | 0,32 | 0,21 | 0,51 | 0,52 | 0,42 | 0,41 | 0,41 | 0,27 |

**Supplementary Table 3.** Sensitivity analysis showing the relationship between the AR model order *p* and signal length *n*, and the performance of the causality measures. Performance is quantified with the Matthew’s Correlation Coefficient averaged over 100 repeated simulations.
