# Supplementary material for: Evaluation of Directed Causality Measures and Lag Estimations in Multivariate Time-Series
Source: Front Syst Neurosci. 2021 Oct 22;15:620338. doi: 10.3389/fnsys.2021.620338 (PMC8569855; doi:10.3389/fnsys.2021.620338)
Supplement: Supplementary file 10 [file Data_Sheet_1.PDF]

1 APPENDIX: TESTING FOR STATISTICAL SIGNIFICANCE

Each causality measure returns a value that quantifies the strength of the detected relationship between the observed variables. In the absence of causal interactions the expected output is zero for each metric, but there is always some variability. Because of this, we must find a suitable threshold that allows to distinguish between non-zero outputs caused by noise or genuine interactions. In this study we compare the ability of several statistical significance tests to correctly classify true relationships, for all combinations of simulation models and causality measures.

Statistical significance can be assessed using parametric tests that have been developed for each measure separately. These are however based on several assumptions that may not hold, and are therefore expected to perform poorly for some simulation models (and also possibly in real measurements). To overcome these limitations we also evaluate the accuracy of non-parametric tests based on surrogate data created using re-sampling techniques in time or frequency domain (block permutation and phase shuffling respectively).

1.1 Parametric statistical tests

Parametric tests have been developed for each causality measure separately, that determine which of the interactions can be considered statistically significant. Since these impose further assumptions on the characteristics of the time series and underlying processes, they add another source of bias and thus possibly erroneous results when these assumptions are not met. In this study we chose the parametric tests as suggested for each measure in literature.

Cross-correlation

The significance test for cross-correlation is based on the large-sample approximation. Let x_t and y_t denote two independent time series with zero mean and unit variance. In this case lag variables x_t and $y_{t+\tau}$ are independent for any selected time point t and for all lag values τ, which means their product $x_t y_{t+\tau}$ is on average zero. Hence, the cross-correlation $\rho_{i,j}(\tau)$ between two independent time series is, correctly, centred around zero. For independent repetitions of independent variables x_t and y_t, the standard deviation of the sample correlation coefficient is approximately equal to $1/\sqrt{n}$, with n the number of observations. The 5% significance limits are:

$$L = \pm \frac{1.96}{n} \tag{1}$$

In strongly autocorrelated signals the pairs (x_t, y_t) are no longer temporally independent and because of this cross-correlation is notorious for returning spurious connections in case of autocorrelated signals. The time series should therefore always be pre-whitened before applying cross-correlation, if significance is tested based on this parametric limit. Here we use a standard pre-whitening procedure based on the Singular Value Decomposition of the covariance matrix.

(Conditional) Granger Causality Index

Statistical significance of the Granger causality-based measures can be checked with an F-test for the null hypothesis that there is no causal relationship between the driver and response variables. In this case, the coefficients of the lagged driving variables are assumed to be zero in the unrestricted[1] AR model. If P_1 and P_2 denote the number of variables in the restricted and unrestricted AR model, respectively, and n the number of observations, then the F-statistic is defined as:

[1] In the case of GCI, unrestricted and restricted refer to the bivariate and univariate AR models respectively. For CGCI these are the AR models of all K variables and of all except the driver, respectively.

$$F = \frac{(RSS_1 - RSS_2)/(P_1 - P_2)}{RSS_2/(n - P_2)} \tag{2}$$

RSS_i is the sum of squares of the residuals from model i. If no (Granger) causal relationship exists between the inspected variables, the unrestricted model does not provide a significantly better fit than the restricted model and the F-statistic follows the F distribution with $(P_2 - P_1, n - P_2)$ degrees of freedom. If the calculated value of the F-statistic exceeds the critical value of the F-distribution for some desired false-rejection probability (here $\alpha = 0.05$), then the null hypothesis is rejected and the found causal relationship is deemed significant.

Partial Directed Coherence / Directed Transfer Function

The critical value for statistical significance of the partial directed coherence again starts from the premise that the autoregressive coefficients $A_{ij}(f)$ are zero under the null hypothesis that there is no Granger causality $X_j \rightarrow X_i$. Computations of the test statistic are a bit more complex and follow a χ^2-distribution. Non-zero PDC values are considered significant, at a given significance level α, if the magnitude $|A_{ij}(f)|$ exceeds the following limit: (Baccala and Sameshima, 2001)

$$c_{PDC}(f) = \sqrt{\frac{\hat{C}_{ij}(f)\chi^2_{1,1-\alpha}}{N\sum_k |\hat{A}_{kj}(f)|^2}} \tag{3}$$

$\chi^2_{1,1-\alpha}$ denotes the $(1 - \alpha)$ quantile of the χ^2 distribution with one degree of freedom, and $\hat{C}_{ij}(f)$ is an estimate of the expression:

$$C_{ij}(f) = \Sigma_{ii}(\sum_{k,l=1}^{P} \Sigma_{jj}^{-1}[cos(kf)cos(lf) + sin(kf)sin(lf)])$$

where Σ_{jj}^{-1} denotes the entries of the inverse of the covariance matrix, Σ, of the VAR process.

A critical value for the directed transfer function can be derived in a similar fashion:

$$c_{DTF}(f) = \sqrt{\frac{\hat{C}_{ij}(f)\chi^2_{1,1-\alpha}}{N\sum_k |\hat{H}_{ik}(f)|^2}} \tag{4}$$

Partial Mutual Information on Mixed Embedding

Because of the way PMIME is calculated, with an internal stopping criterion that checks at each iteration if the lagged variable holds a significant amount of additional information for the embedding vector, it does not rely on any further significance testing. It returns zero in case of uncoupled variables and positive non-zero values otherwise. This was confirmed using time-shifted surrogates, and the PMIME values of the surrogate time series were all zero (Papana et al., 2013).

1.2 Block permutation

Because of the underlying assumptions of parametric significance tests are not always met, the resulting conclusions on causal relationships may be unreliable. Another way to test for significance that mitigates this issue is by generating surrogate data. The aim is to create a large set of artificial time series datasets,

based on the original signals, wherein any possible causal influence between the variables is destroyed whilst preserving as much as possible the other characteristics of the signals. One of the assumptions behind causality is that the cause always precedes a change in value of its effect. By randomly permuting the signals in time domain, this temporal relationship between the driver and response variables can be removed.

With the block permutation method, each signal is cut into a number of chunks of equal size that are randomly permuted to create M surrogate time series that are consistent with the non-causality null hypothesis (i.e. H_0: X_2 does not Granger cause X_1). The number of blocks into which the signal is divided will obviously impact the results and should be optimised. If the blocks are too short the dynamics of the time series will be affected too much, while large block lengths will fail to adequately remove the causal relationships. For time series length $n = 1000$, we found a permutation scheme with 10 blocks (i.e. block length = 100 samples) to be optimal for the causality measures considered here.

An empirical null distribution can now be constructed by computing the causality measure on the M surrogate time series. Since the output values are assumed to be zero for all measures in the absence of causality, a one-sided rank test can be used to evaluate the significance of the original causality measure value. If the estimate is at the tail of the empirical null distribution, then H_0 is rejected. If r is the rank of the original estimate of the causality measure, the p-value is $p = 1 - r/(n_{surr} + 1)$, with n_{surr} the number of surrogate datasets. Here we use $n_{surr} = 100$.

1.3 Time-shift surrogates

Because block permutation surrogates suffer from edge effects, we include another surrogate technique called time-shifting. By shifting each time series with a random delay the coupling between the signals is destroyed, while the signal characteristics are preserved. Same as in the block permutation method, this process is repeated multiple times to get an empirical null distribution against which the actual connectivity strength value can be tested.

1.4 Phase shuffling

The second method that's used in this study to generate surrogate data involves shuffling the signal phases. Because phase changes in the frequency domain are associated with temporal translations in time domain (and vice versa), changing the phase of a signal has a similar effect as creating time-shifted surrogate data and can effectively destroy temporal relationships between time series. Compared to sample shuffling, and in lesser extent also block permutation, phase shuffling has the advantage of preserving the spectral distribution of the original signal and hence creates surrogate time series with dynamics that better approach the original signals. The phase shuffled surrogate data is created by taking the signal to the frequency domain using the discrete Fourier transform, and then assigning random phase values (taken uniformly from the range $[0, 2\pi]$) to each spectral component. With the inverse Fourier transform the signal is taken back to the time domain and the surrogate time series are obtained. If the surrogate data needs to be a real signal (i.e. not complex), one should take care that the phases are made anti-symmetric before applying the inverse Fourier transform.

1.5 Results

Here we investigate the impact on the performance of the different sig nificance tests being parametric tests, block permutation, time-shifting and phase shuffling. Figure 1 shows two bar graphs with the average MCC values for each significance test, where the average is taken over the simulation models or the causality measures. The performance of cross-correlation is significantly lower than the other measures. As this might skew the results, cross-correlation values were left out when computing the average MCC over the causality measures. Looking a figure 1A, the surrogate techniques outperform the parametric tests

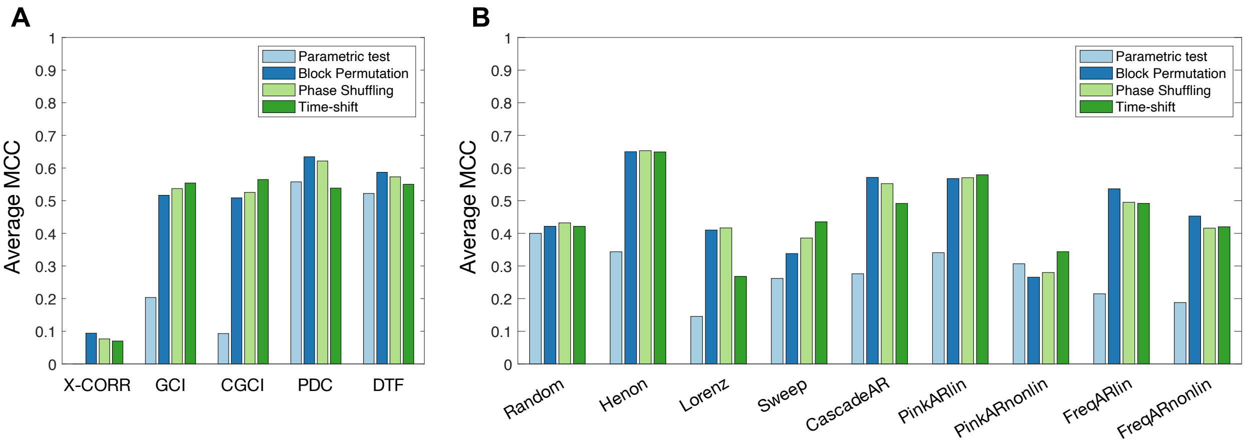

Figure 1. Comparison of the MCC values for the different significance tests, averaged over (**A**) the simulation models, or (**B**) the causality measures.

when combined with GCI and CGCI. For PDC and DTF the results are more similar. In figure 1B we see the same general trend where significance testing based on surrogate data seems to be more robust over the different model types and give similar or slightly better results than with parametric tests. The average MCC values are within the same range for all surrogate techniques.

Note that PMIME is left out of this comparison, as surrogate data testing takes an excessive amount of time for this measure and it has already been shown that it doesn't need any further significance testing (see section 1.1). The MCC values for all possible combinations of simulation mode, causality measure and significance test are summarised in table 1.

	XCORR				GCI				CGCI				PDC				DTF			
	Stat	PB	TS	PS	Stat	PB	TS	PS	Stat	PB	TS	PS	Stat	PB	TS	PS	Stat	PB	TS	PS
Random	0,00	0,18	0,01	0,18	0,00	0,69	0,74	0,72	0,00	0,32	0,38	0,32	1,00	0,50	0,52	0,52	1,00	0,42	0,46	0,42
Henon	0,00	0,03	0,00	0,00	0,18	0,75	0,74	0,78	0,17	0,83	0,84	0,84	0,88	0,86	0,86	0,86	0,48	0,78	0,81	0,79
Lorenz	0,00	0,18	0,15	0,17	0,22	0,44	0,27	0,47	0,11	0,41	0,30	0,44	0,20	0,53	0,33	0,52	0,20	0,49	0,29	0,48
Sweep	0,00	-0,08	-0,09	-0,09	0,15	0,45	0,68	0,48	-0,13	0,30	0,43	0,33	0,91	0,55	0,41	0,62	0,38	0,48	0,75	0,59
CascadeAR	0,00	-0,17	-0,18	-0,18	0,28	0,62	0,54	0,60	0,02	0,87	0,92	0,85	0,53	0,91	0,75	0,89	0,54	0,63	0,43	0,59
PinkARlin	0,00	0,25	0,23	0,24	0,25	0,29	0,42	0,30	0,29	0,58	0,88	0,61	0,56	0,87	0,65	0,86	0,61	0,85	0,71	0,84
PinkARnonlin	0,00	0,26	0,39	0,25	0,25	0,31	0,41	0,35	0,26	0,14	0,25	0,21	0,59	0,28	0,29	0,26	0,44	0,34	0,38	0,32
FreqARLin	0,00	0,11	0,07	0,08	0,25	0,59	0,64	0,60	0,05	0,59	0,56	0,58	0,23	0,67	0,56	0,57	0,54	0,72	0,63	0,65
FreqARnonlin	0,00	0,09	0,04	0,03	0,25	0,51	0,55	0,52	0,05	0,53	0,51	0,54	0,13	0,56	0,49	0,50	0,51	0,58	0,50	0,48

Table 1. MCC values for each combination of simulation model, causality measure and significance test. The statistical significance of the connections is evaluated using the parametric test (Stat), block permutation (PB), time-shift (TS) and phase shuffling (PS) tests.

1.6 Discussion

We evaluated the influence of the using parametric or surrogate techniques for testing the significance of connectivity strength values. We compared four testing methods: parametric tests, block permutation, time-shifting and phase shuffling. During this evaluation it already becomes apparent that cross-correlation is unable to reliably capture the dynamics of the different simulated systems and is significantly outperformed by the other, more advanced, measures. The performance of the significance tests varies when looking over the different causality measures and simulation models. Parametric tests seem to perform competitively with the surrogate techniques for the simplest simulation model ('Random'), but is outperformed by surrogate testing for the more complex (and more realistic) models. This trend was expected, since one of

the main advantages of using surrogate data is that it's independent of the causality measure and relies on less mathematical assumptions. The results for block permutation, time-shifting and phase shuffling are comparable, and they all seem to be fitting candidates to use in this study. Because phase shuffling shows a slightly lower variability in performance, we decided to use this technique to assess statistical significance of the connections.
